# Effects of MAO-B inhibitors on non-motor symptoms and quality of life in Parkinson’s disease: A systematic review

**DOI:** 10.1038/s41531-022-00339-2

**Published:** 2022-06-13

**Authors:** Takashi Tsuboi, Yuki Satake, Keita Hiraga, Katsunori Yokoi, Makoto Hattori, Masashi Suzuki, Kazuhiro Hara, Adolfo Ramirez-Zamora, Michael S. Okun, Masahisa Katsuno

**Affiliations:** 1 Department of Neurology, Nagoya University Graduate School of Medicine, Nagoya, Japan; 2 Department of Neurology, Norman Fixel Institute for Neurological Diseases, University of Florida, Gainesville, FL USA; 3 Department of Neurology, National Center for Geriatrics and Gerontology, Obu, Japan; 4 Department of Clinical laboratory, Nagoya University Hospital, Nagoya, Japan

**Keywords:** Parkinson's disease, Parkinson's disease

## Abstract

Non-motor symptoms (NMS) are common among patients with Parkinson’s disease and reduce patients’ quality of life (QOL). However, there remain considerable unmet needs for NMS management. Three monoamine oxidase B inhibitors (MAO-BIs), selegiline, rasagiline, and safinamide, have become commercially available in many countries. Although an increasing number of studies have reported potential beneficial effects of MAO-BIs on QOL and NMS, there has been no consensus. Thus, the primary objective of this study was to provide an up-to-date systematic review of the QOL and NMS outcomes from the available clinical studies of MAO-BIs. We conducted a literature search using the PubMed, Scopus, and Cochrane Library databases in November 2021. We identified 60 publications relevant to this topic. Overall, rasagiline and safinamide had more published evidence on QOL and NMS changes compared with selegiline. This was likely impacted by selegiline being introduced many years prior to the field embarking on the study of NMS. The impact of MAO-BIs on QOL was inconsistent across studies, and this was unlikely to be clinically meaningful. MAO-BIs may potentially improve depression, sleep disturbances, and pain. In contrast, cognitive and olfactory dysfunctions are likely unresponsive to MAO-BIs. Given the paucity of evidence and controlled, long-term studies, the effects of MAO-BIs on fatigue, autonomic dysfunctions, apathy, and ICD remain unclear. The effects of MAO-BIs on static and fluctuating NMS have never been investigated systematically. More high-quality studies will be needed and should enable clinicians to provide personalized medicine based on a non-motor symptom profile.

## Introduction

Three monoamine oxidase B inhibitors (MAO-BIs) are now commercially available in many countries for the management of motor symptoms in patients with Parkinson’s disease (PD). Selegiline and rasagiline are irreversible MAO-BIs, while safinamide is a reversible MAO-BI^1^. These MAO-BIs possess distinct pharmacological profiles (i.e., potency, MAO-B/MAO-A selectivity, and pharmacokinetics). In addition, safinamide modulates voltage-sensitive sodium and calcium channels activity and reduces glutamate release^2,3^.

The results of large clinical trials have been reported since the 1990s for selegiline, since the 2000s for rasagiline, and since the 2010s for safinamide. Notably, selegiline was largely studied before the field embarked on defining non-motor symptoms (NMS) of PD and developing specific and applicable scales. Recognition of NMS has also evolved over recent years^4,5^. Many double-blind, placebo-controlled randomized controlled studies (RCTs) revealed the beneficial effects of MAO-BIs on motor symptoms and wearing-off compared with placebo^1,6,7^. These findings are corroborated by meta-analyses^8–10^. The superiority of one MAO-BI over others remains undetermined because there have been no high-quality direct comparative trials among the MAO-BIs.

NMS of PD include depression, anxiety, sleep disturbances, fatigue, pain, and cognitive and autonomic dysfunctions, and underpin the entire course from the prodromal to late stage^5,11^. Past studies revealed that NMS were more relevant than motor symptoms in quality of life (QOL)^12,13^. A review on level 1 evidence for treatment of NMS by the Movement Disorders Society was published in 2019 and suggested that there remain considerable unmet needs for NMS management^5^. Although an increasing number of MAO-BI studies have reported QOL or NMS outcomes, to the best of our knowledge, no reviews have systematically summarized those results. Thus, this systematic review aimed (1) to summarize QOL and NMS outcomes from clinical studies of MAO-BIs, (2) to guide clinicians to select MAO-BIs based on a patient’s symptom profile, and (3) to facilitate future investigations on these issues.

## Results

### Literature search

**Fig. 1:**
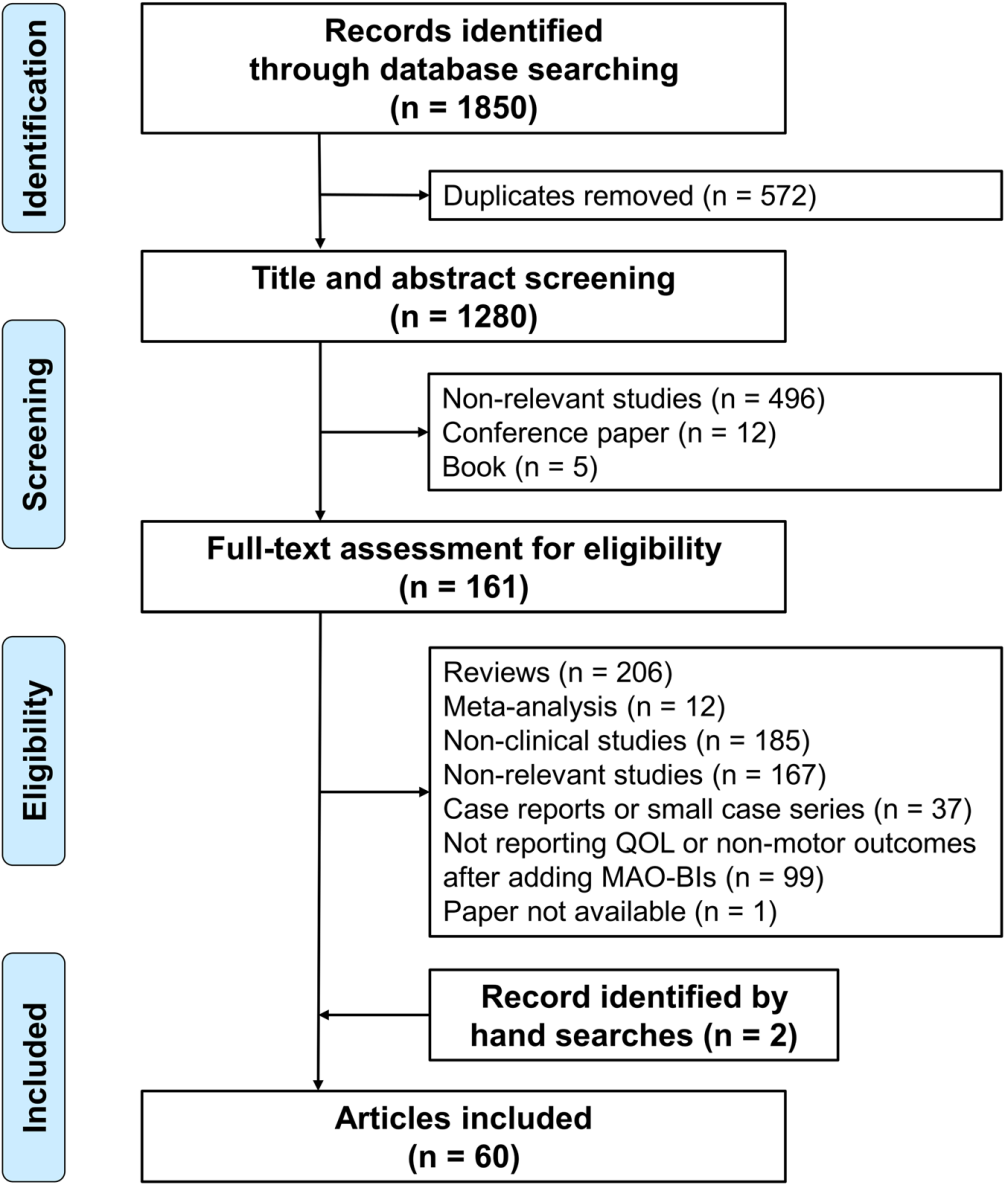
Flowchart of the literature search. A systematic literature search using the PubMed, Scopus, and Cochrane Library databases was conducted.

The systematic literature search revealed 1850 records (Fig. 1). By performing duplicate removal, title/abstract screening, full-text assessments, and hand searches, we identified 60 clinical studies which met the eligibility criteria. Most studies enrolled either early PD patients or advanced PD patients experiencing wearing-off, whereas a minority of studies focused on specific populations: patients with sleep disturbances (three studies)^14–16^, depression (two studies)^17,18^, mild cognitive impairment (MCI) (two studies)^19,20^, freezing of gait (two studies)^21,22^, fatigue (one study)^23^, urinary symptoms (one study)^24^, high non-motor burden (one study)^25^, or RBD (one study)^26^. There are only five double-blind, placebo-controlled RCTs that investigated non-motor outcomes as the primary outcomes (rasagiline for MCI^19,20^, rasagiline for depression^17^, rasagiline for fatigue^23^, and rasagiline for sleep disturbances)^14^. The remaining studies are RCTs reporting QOL or non-motor results as the secondary outcomes or open-label studies. In the following paragraphs, QOL and NMS outcomes in the literature will be systematically summarized along with Tables 1–7.

**Table 1:** Quality of life outcomes of MAO-BI studies.

Studies	Study design	Participants	Study quality	Age	Disease duration	Instruments	Outcome	Effect size
Parkinson study group (2005)^33^	Multicenter, double-blind, placebo-controlled RCT, 26 weeks	472 patients, advanced PD with off time ≥ 2.5 h	1	63.3 (9.5)	9.3 (5.3)	PDQUALIF scale	No significant difference between rasagiline 1 mg and placebo, −1.48 (−3.86 to 0.90), *p* = 0.22	IC
						PDQUALIF scale	No significant difference between rasagiline 0.5 mg and placebo −2.18 (−4.49 to 0.14), *p* = 0.07	IC
Parkinson study group (2002)^34^	Multicenter, double-blind, placebo-controlled RCT, 26 weeks	404 patients, early PD not requiring dopaminergic therapy	1	60.8 (10.8)	1.0 (1.2)	PDQUALIF scale	Significantly better in rasagiline 1 mg vs placebo, −2.91 (−5.19 to −0.64), *P* < 0.05	IC
						PDQUALIF scale	Significantly better in rasagiline 2 mg vs placebo, −2.74 (−5.02 to −0.45), *P* < 0.05	IC
Hattori et al. (2018)^31^	Multicenter, double-blind, placebo-controlled RCT, 26 weeks	404 patients, advanced PD with off time ≥ 2.5 h	1	66.1 (8.3)	9.0 (4.7)	PDQ-39	Significantly better in rasagiline 1 mg vs placebo, −3.84 (−6.16 to −1.52), *p* = 0.0012	0.08
						PDQ-39	Significantly better in rasagiline 0.5 mg vs placebo, −2.51 (−4.79 to −0.23), *p* = 0.0309	0.03
Zang et al. (2018)^32^	Multicenter, double-blind, placebo-controlled RCT, 16 weeks	324 patients, advanced PD with off time ≥ 1 h	1	62.2 (9.4)	7.3 (4.6)	EQ-5D	Significantly better in rasagiline 1 mg vs placebo, 0.05 (0.01 to 0.09), *p* = 0.024	IC
						EQ-5D: visual analog scale	Significantly better in rasagiline 1 mg vs placebo, 4.31 (1.18 to 7.45), *p* = 0.007	IC
						PDQ-39	No significant difference between rasagiline 1 mg and placebo, −1.8 (−3.96 to 0.42), *p* = 0.1122	IC
Hauser et al. (2014)^28^	Multicenter, double-blind, placebo-controlled RCT, 18 weeks	321 patients, early PD not adequately controlled with dopamine agonizts	1	62.6 (9.7)	2.1 (2.1)	PDQ-39	No significant differences between rasagiline 1 mg and placebo; statistics not shown	IC
Hattori et al. (2019)^29^	Multicenter, double-blind, placebo-controlled RCT, 26 weeks	244 early PD patients not taking antiparkinsonian medication	1	66.4 (8.9)	1.8 (1.6)	PDQ-39	No significant differences between rasagiline 1 mg and placebo; −1.60 (−3.59 to 0.38), *P* = 0.1128	0.16
Hattori et al. (2019)^35^	Open-label extension of a multicenter, double-blind, placebo-controlled RCT, 52 weeks	198 early PD patients not taking antiparkinsonian medication	1	66.5 (9.1)	1.8 (1.7)	PDQ-39	Significant worsening with Rasagiline at 52 weeks; baseline to post 2.86 (1.29 to 4.43), *P* value not shown	0.28
						PDQ-39	Significant improvement with placebo 26 weeks and rasagiline 26 weeks; baseline to post −1.50 (−2.85 to −0.15), *P* value not shown	0.14
Zhang et al. (2018)^30^	Multicenter, double-blind, placebo-controlled RCT, 26 weeks	130 early PD patients not taking antiparkinsonian medication	1	59.0 (8.9)	0.1 (median)	PDQ-39	No significant differences between groups; rasagiline 1 mg −0.77 ± 1.12 vs. placebo 1.97 ± 1.15, *P* = 0.425	IC
						EQ-5D	No significant differences between groups; rasagiline 1 mg −0.01 ± 0.02 vs. placebo −0.04 ± 0.02, *P* = 0.261	IC
						EQ-5D: visual analog scale	Significantly better in rasagiline; rasagiline 1 mg 2.49 ± 1.61 vs. placebo −4.31 ± 1.65, *P* = 0.002	IC
Barone et al. (2015)^17^	Multicenter, double-blind, placebo-controlled RCT, 12 weeks	123 patients, PD with moderate depression (BDI ≥ 15)	1	66.1 (8.5)	4.3 (12.5)	PDQ-39	No significant difference between groups, rasagiline 1 mg −6.24 ± 2.69 vs. placebo −1.03 ± 2.33, *P* = 0.074	0.27
Lim et al. (2015)^23^	Multicenter, double-blind, placebo-controlled RCT, 12 weeks	30 patients, PD with moderate to severe fatigue (FSS ≥ 4)	1	68.7 (7.4)	3 (median)	PDQ-39	Significantly better in rasagiline 1 mg vs placebo (19 vs -6 points), *P* = 0.018	IC
Schrempf et al. (2018)^14^	Single-center, double-blind, placebo-controlled RCT, 8 weeks	20 patients, PD with sleep disturbances (PSQI > 5)	1	69.9 (6.9)	4.0 (3.5)	PDQ-39	No significant change with rasagiline 1 mg; baseline 30.4 ± 19.3 and post 29.4 ± 22.9, *p* = 0.686	0.05
Hattori et al. (2019)^36^	Multicenter, open-label, prospective, phase 3 study, 52 weeks	222 PD patients taking levodopa with or without motor fluctuation	3	68.0 (8.4)	7.1 (5.0)	PDQ-39	No significant change with rasagiline 1 mg; baseline to post −0.64 ± 9.41, *P* value not shown	0.05
Cibulcik et al. (2016)^21^	Single-center, open-label, prospective study, 3 months	42 patients, PD with freezing of gait	3	69.5 (7.9)	8.3 (4.3)	PDQ-39	Significant improvement with rasagiline 1 mg; baseline 31.4 ± 13.2 and post 28.7 ± 14.7, *p* < 0.001	0.19
Müller et al. (2013)^15^	Single-center, open-label, prospective study, 4 months	30 patients, PD with sleep disturbances	3	66.6 (6.5)	NA	PDQ-39	Not significantly changed after switching selegiline 7.5 mg to rasagiline 1 mg; baseline 24.6 ± 2.8 to 22.6 ± 2.6, *P* value not shown	0.13
Borgohain et al. (2014)^37^	Multicenter, double-blind, placebo-controlled RCT, 24 weeks	669 patients, advanced PD with off time > 1.5 h	1	59.9 (9.4)	8.1 (3.9)	PDQ-39	Significantly better in safinamide; safinamide 100 mg −28.4 vs. placebo −11.9, *P* = 0.0360	0.23
						PDQ-39	No significant differences between groups; safinamide 50 mg −16.4 vs. placebo −11.9, *P* = 0.5603	0.15
Schapira et al. (2017)^38^	Multicenter, double-blind, placebo-controlled RCT, 24 weeks	549 patients, advanced PD with off time > 1.5 h	1	61.9(9.0)	8.9 (4.6)	EQ-5D	Significantly better in safinamide; safinamide 100 mg 0.03 ± 0.19 vs. placebo −0.03 ± 0.19, *P* < 0.001	0.17
						PDQ-39	Significantly better in safinamide; safinamide 100 mg −3.17 ± 10.86 vs. placebo −0.68 ± 10.51, *P* = 0.006	0.22
Borgohain et al. (2014)^39^	Multicenter, double-blind, placebo-controlled RCT, 2 years	544 patients, advanced PD with off time > 1.5 h	1	59.9 (9.4)	8.1 (3.9)	PDQ-39	Significant improvement with safinamide 100 mg vs placebo; statistics not shown	IC
						PDQ-39	No significant improvement with safinamide 50 mg vs placebo; statistics not shown	IC
Hattori et al. (2020)^40^	Multicenter, double-blind, placebo-controlled RCT, 24 weeks	406 patients, advanced PD with wearing off	1	68.1 (8.6)	8.2 (4.9)	PDQ-39	No significant differences between groups; safinamide 50 mg −1.70 ± 0.84 vs. placebo −1.37 ± 0.86, *P* = 0.783	0.11
						PDQ-39	No significant differences between groups; safinamide 100 mg −3.38 ± 0.85 vs. placebo −1.37 ± 0.86, *P* = 0.097	0.23
Cattaneo et al. (2020)^41^	Post-hoc analysis of a multicenter, double-blind, placebo-controlled RCT, 2 years	352 patients, advanced PD with off time > 1.5 h	1	NA	NA	PDQ-39	Significantly better in safinamide 100 mg vs placebo, −2.44 (−4.75 to −0.12), *P* = 0.0390	IC
Tsuboi et al. (2020)^42^	Multicenter, open-label, prospective study, 52 weeks	203 patients, advanced PD with wearing off	3	67.2 (8.6)	9.8 (5.3)	PDQ-39	No significant change with safinamide 50 or 100 mg; baseline to post −0.85 ± 0.90, *P* value not shown	0.06
Santos García et al. (2021)^25^	Multicenter, open-label, prospective study, 6 months	50 patients, PD with high non-motor burden (NMSS ≥ 40)	3	68.5 (9.1)	6.4 (5.1)	PDQ-39	Significant improvement with safinamide 100 mg; baseline 30.1 ± 17.6 and post 21.2 ± 13.5, *P* < 0.0001	0.50
Grigoriou et al. (2021)^43^	Multicenter, open-label, prospective study, 6 months	27 patients, advanced PD with off time > 1.5 h	3	65	6.8	PDQ-8	No significant change with safinamide 100 mg; baseline 30.1 ± 18.1 and post 30.1 ± 18.3, *p* = 0.89	0.00
						EQ-5D	No significant change with safinamide 100 mg; baseline 0.67 ± 0.23 and post 0.72 ± 0.19, *p* = 0.22	0.22
De Micco et al. (2021)^44^	Single-center, open-label, prospective study, 6 months	20 patients, advanced PD with off time > 1.5 h	3	63.8 (10.2)	6.0 (2.2)	PDQ-39	No significant change with safinamide 50 mg; baseline 43.1 ± 7.41 and post 30.4 ± 23.6, *P* = 0.25	0.34
Bianchi et al. (2019)^45^	Single-center, open-label, retrospective study, 4.4 months	20 patients, advanced PD with motor fluctuations	4	75.0 (6.3)	14.5 (6.8)	PDQ-8	Significant improvement with safinamide 100 mg; baseline 9.4 ± 5.4 and post 5.0 ± 5.7, P = 0.04	0.81
						EQ-5D	No significant change with safinamide 100 mg; baseline 7.7 ± 2.0 and post 6.5 ± 2.0, *P* = 0.10	0.60
						EQ-5D: visual analog scale	No significant change with safinamide 100 mg; baseline 65.0 ± 16.2 and post 71.0 ± 19.8, *P* = 0.40	0.37
Geroin et al. (2020)^46^	Single-center, open-label, prospective study, 12 weeks	13 patients, advanced PD with motor fluctuation and pain (NRS ≥ 4)	3	64.1 (6.7)	5.8 (2.9)	PDQ-39	Significant improvement with safinamide 100 mg; baseline to post −11.2 ± 6.7, *P* < 0.05	IC

Age and disease duration are presented as mean (SD) if available.

*BDI* Beck Depression Inventory, *EQ-5D* EuroQol 5 Dimension, *FSS* Fatigue Severity Scale, *IC* incalculable, *NA* not assessed, *NMSS* Non-Motor Symptoms Scale, *NRS* Numeric Rating Scale, *PD* Parkinson’s Disease, *PDQ* Parkinson’s Disease Questionnaire, *PSQI* Pittsburgh Sleep Quality Index, *RCT* Randomized Controlled Trial.

**Table 2:** Depression and anxiety outcomes of MAO-BI studies.

Studies	Study design	Participants	Study quality	Age	Disease duration	Instruments	Outcome	Effect size
Parkinson study group (2002)^34^	Multicenter, double-blind, placebo-controlled RCT, 26 weeks	404 patients, early PD not requiring dopaminergic therapy	1	60.8 (10.8)	1.0 (1.2)	BDI	No significant difference between rasagiline 1 mg and placebo, −0.35 (−0.86 to 0.16), *P* > 0.05	IC
						BDI	No significant difference between rasagiline 2 mg and placebo, −0.21 (−0.72 to 0.30), *P* > 0.05	IC
Hattori et al. (2018)^31^	Multicenter, double-blind, placebo-controlled RCT, 26 weeks	404 patients, advanced PD with off time ≥ 2.5 h	1	66.1 (8.3)	9.0 (4.7)	PDQ-39: emotional well-being	Significantly better in rasagiline 1 mg vs placebo, −4.10 (−7.81 to −0.39), *p* = 0.0303	IC
						PDQ-39: emotional well-being	Significantly better in rasagiline 0.5 mg vs placebo, −3.76 (−7.41 to −0.11), *p* = 0.0434	IC
Zang et al. (2018)^32^	Multicenter, double-blind, placebo-controlled RCT, 16 weeks	324 patients, advanced PD with off time ≥ 1 h	1	62.2 (9.4)	7.3 (4.6)	PDQ-39: emotional well-being	No significant difference between rasagiline 1 mg and placebo, −2.4 (−6.35 to 1.49), *p* = 0.224	IC
Hattori et al. (2019)^29^	Multicenter, double-blind, placebo-controlled RCT, 26 weeks	244 early PD patients not taking antiparkinsonian medication	1	66.4 (8.9)	1.8 (1.6)	PDQ-39: emotional well-being	Significantly better in rasagiline 1 mg vs placebo; −3.70 (−6.67 to −0.72), *P* = 0.0150	IC
Zhang et al. (2018)^30^	Multicenter, double-blind, placebo-controlled RCT, 26 weeks	130 early PD patients not taking antiparkinsonian medication	1	59.0 (8.9)	0.1 (median)	PDQ-39: emotional well-being	No significant differences between groups; rasagiline 1 mg −1.62 ± 1.78 vs. placebo 1.43 ± 1.82, *P* = 0.201	IC
Barone et al. (2015)^17^	Multicenter, double-blind, placebo-controlled RCT, 12 weeks	123 patients, PD with moderate depression (BDI ≥ 15)	1	66.1 (8.5)	4.3 (12.5)	BDI	No significant difference between groups at 12 weeks; rasagiline 1 mg −5.40 ± 0.79 vs. placebo −4.43 ± 0.73. *P* = 0.368	1.01
						BDI	Significantly better in rasagiline at 4 weeks; rasagiline 1 mg −5.46 ± 0.73 vs. placebo –3.22 ± 0.67, *P* = 0.026	1.02
						PDQ-39: emotional well-being	No significant difference between groups, rasagiline 1 mg −5.66 ± 2.54 vs. placebo −2.33 ± 2.23, *P* value not shown	0.28
Stern et al. (2004)^47^	Multicenter, double-blind, placebo-controlled RCT, 10 weeks	56 early PD patients not taking antiparkinsonian medication	1	61.5 (8.8)	0.7 (1.5)	BDI	No significant difference between rasagiline and placebo, statistics not shown	IC
Hanagasi et al. (2011)^19^	Multicenter, double-blind, placebo-controlled RCT, 12 weeks	55 patients, mild to moderate PD (HY stage 1–3) with mild cognitive impairment	1	66.4 (9.8)	4.0 (2.4)	Geriatric depression scale	No significant differences between rasagiline 1 mg and placebo; −0.16 ± 1.37, *P* = 0.86	0.12
						Anxiety-state score	No significant differences between rasagiline 1 mg and placebo; −3.37 ± 2.3, *P* = 0.164	0.22
						Anxiety-trait score	No significant differences between rasagiline 1 mg and placebo; −2.11 ± 1.96, *P* = 0.288	0.10
Lim et al. (2015)^23^	Multicenter, double-blind, placebo-controlled RCT, 12 weeks	30 patients, PD with moderate to severe fatigue (FSS ≥ 4)	1	68.7 (7.4)	3 (median)	State-trait anxiety inventory	No significant differences between rasagiline 1 mg and placebo (12.5 vs 5.5 points), *P* = 0.30	IC
						BDI-II	Significantly better in rasagiline 1 mg vs placebo (5.5 vs 0.5 points), *P* = 0.018	IC
Hattori et al. (2019)^36^	Multicenter, open-label, prospective, phase 3 study, 52 weeks	222 PD patients taking levodopa with or without motor fluctuation	3	68.0 (8.4)	7.1 (5.0)	PDQ-39: emotional well-being	No significant change with rasagiline 1 mg; baseline to post 0.37 ± 14.83, *P* value not shown	IC
Cibulcik et al. (2016)^21^	Single-center, open-label, prospective study, 3 months	42 patients, PD with freezing of gait	3	69.5 (7.9)	8.3 (4.3)	PDQ-39: emotional well-being	No significant change with rasagiline 1 mg; baseline 21.8 ± 14.5 and post 19.5 ± 14.5, *p* = 0.099	0.16
Müller et al. (2013)^15^	Single-center, open-label, prospective study, 4 months	30 patients, PD with sleep disturbances	3	66.6 (6.5)	NA	HAMD	Significantly improved after switching selegiline 7.5 mg to rasagiline 1 mg; baseline −8.1 ± 0.6 to −6.9 ± 0.7, *P* = 0.003	0.37
Rahimi et al. (2016)^22^	Single-center, open-label, prospective study, 90 days	14 patients, PD with freezing of gait	3	68.9 (6.7)	11.8 (5.0)	Beck anxiety inventory	No significant change with rasagiline 1 mg; mean values for the whole cohort not shown, P = 0.80	IC
						BDI	No significant change with rasagiline 1 mg; mean values for the whole cohort not shown, P = 0.22	IC
Borgohain et al. (2014)^37^	Multicenter, double-blind, placebo-controlled RCT, 24 weeks	669 patients, advanced PD with off time > 1.5 h	1	59.9 (9.4)	8.1 (3.9)	PDQ-39: emotional well-being	Significantly better in safinamide; safinamide 100 mg −5.1 vs. placebo −1.7, *P* = 0.0116	0.27
			1	59.9 (9.4)	8.1 (3.9)	PDQ-39: emotional well-being	No significant differences between groups; safinamide 50 mg −2.4 vs. placebo −1.7, *P* = 0.6123	0.12
			1	59.9 (9.4)	8.1 (3.9)	GRID-HAMD	No significant differences between groups; safinamide 100 mg −0.8 vs. placebo 0.3, *P* = 0.0731	0.23
			1	59.9 (9.4)	8.1 (3.9)	GRID-HAMD	No significant differences between groups; safinamide 50 mg −0.5 vs. placebo −0.3 *P* = 0.3922	0.14
Schapira et al. (2017)^38^	Multicenter, double-blind, placebo-controlled RCT, 24 weeks	549 patients, advanced PD with off time > 1.5 h	1	61.9(9.0)	8.9 (4.6)	GRID-HAMD	No significant differences between groups; safinamide 100 mg 0.07 ± 3.61 vs. placebo 0.32 ± 4.11, *P* = 0.32	0.02
Borgohain et al. (2014)^39^	Multicenter, double-blind, placebo-controlled RCT, 2 years	544 patients, advanced PD with off time > 1.5 h	1	59.9 (9.4)	8.1 (3.9)	GRID-HAMD	Significant improvement with safinamide 100 mg vs placebo; statistics not shown	IC
						GRID-HAMD	No significant improvement with safinamide 50 mg vs placebo; statistics not shown	IC
Cattaneo et al. (2017)^48^	Post-hoc analysis of two multicenter, double-blind, placebo-controlled RCTs, 6 and 24 months	446 patients, advanced PD with off time > 1.5 h	1	NA	NA	PDQ-39: emotional well-being	At 6 months, significantly better in safinamide 100 mg vs placebo; −3.77 (−6.49 to −1.05), *P* = 0.0067	IC
						PDQ-39: emotional well-being	At 24 months, significantly better in safinamide 100 mg vs placebo; −4.66 (−7.30 to −2.02), *P* = 0.0006	IC
						GRID-HAMD	At 6 months, significantly better in safinamide 100 mg vs placebo; −0.57 (−1.13 to −0.02), *P* = 0.0408	IC
						GRID-HAMD	At 24 months, significantly better in safinamide 100 mg vs placebo; −0.87 (−1.44 to −0.30), *P* = 0.0027	IC
Stocchi et al. (2012)^64^	Multicenter, double-blind, placebo-controlled RCT, 24 weeks	269 patients, early PD receiving a stable dose of a single dopamine agonist	1	57.4 (11.3)	2.5 (1.3)	HAMD	No significant difference between safinamide and placebo; statistical values not shown	IC
Schapira et al. (2013)^65^	Multicenter, double-blind, placebo-controlled RCT, 18 months	227 patients, early PD taking a single dopamine agonist	1	median 56.6 and 59.8 for 100 mg and 200 mg	NA	HAMD	No significant differences between groups; safinamide 100 or 200 mg −0.5 ± 3.42 vs. placebo −0.3 ± 2.54, *P* = 0.389	0.15
Peña et al. (2021)^18^	Multicenter, open-label, retrospective study, 3 months	82 patients, PD with depressive symptoms (HAMD-17 > 14)	4	68.3 (11.4)	8.7 (8.6)	HAMD-17	Significant improvement with safinamide 50 mg; baseline to post −4.7 ± 4.5, *P* < 0.0001	1.76
						HAMD-17	Significant improvement with safinamide 100 mg; baseline to post −8.0 ± 5.7, *P* < 0.0001	1.82
Santos García et al. (2021)^25^	Multicenter, open-label, prospective study, 6 months	50 patients, PD with high non-motor burden (NMSS ≥ 40)	3	68.5 (9.1)	6.4 (5.1)	BDI-II	Significant improvement with safinamide 100 mg; baseline 15.9 ± 10.5 and post 10.2 ± 6.8, *P* < 0.0001	0.54
						PDQ-39: emotional well-being	Significant improvement with safinamide 100 mg; baseline 44.3 ± 29.3 and post 26.3 ± 23.0, *P* < 0.0001	0.61
Grigoriou et al. (2021)^43^	Multicenter, open-label, prospective study, 6 months	27 patients, advanced PD with off time > 1.5 h	3	65	6.8	HADS: anxiety	No significant change with safinamide 100 mg; baseline 5.2 ± 3.7 and post 4.8 ± 2.9, *p* = 0.50	0.11
						HADS: depression	No significant change with safinamide 100 mg; baseline 5.0 ± 4.0 and post 4.8 ± 2.9, *p* = 0.70	0.08
De Micco et al. (2021)^44^	Single-center, open-label, prospective study, 6 months	20 patients, advanced PD with off time > 1.5 h	3	63.8 (10.2)	6.0 (2.2)	BDI	No significant change with safinamide 50 mg; baseline 6.90 ± 5.05 and post 6.70 ± 5.93, *P* = 0.91	0.04
						PD Anxiety Scale	No significant change with safinamide 50 mg; baseline 12.0 ± 8.62 and post 10.7 ± 6.87, *P* = 0.59	0.16
Bianchi et al. (2019)^45^	Single-center, open-label, retrospective study, 4.4 months	20 patients, advanced PD with motor fluctuations	4	75.0 (6.3)	14.5 (6.8)	HADS	No significant change with safinamide 100 mg; baseline 10.1 ± 7.1 and post 5.4 ± 5.3, *P* = 0.05	0.66
Shoulson et al. (1992)^51^	Multicenter, double-blind, placebo-controlled RCT, 3 months	800 patients, early PD not taking antiparkinsonian medication	1	61.1 (9.5)	NA	HAMD	Significantly better in selegiline; selegiline 10 mg, baseline 2.8 ± 3.0 and post 2.5 ± 3.0, placebo or tocopherol, baseline 2.59 ± 2.9 and post 2.96 ± 3.81, *P* = 0.0028	0.10
Pålhagen et al. (2006)^52^	Multicenter, double-blind, placebo-controlled RCT, 7 years	140 patients, early de novo PD	1	63.4 (8.1)	3.0 (2.1)	HAMD	Significantly better in selegiline 10 mg than placebo; mean values not shown, *P* = 0.016	IC
Allain et al. (1991)^53^	Multicenter, double-blind, placebo-controlled RCT, 3 months	93 patients, early de novo PD	1	64.9 (9.3)	NA	HAMD	Significantly better in selegiline; selegiline 10 mg, baseline 6.0 ± 4.5 and post 3.0 ± 3.4, placebo, baseline 6.0 ± 5.0 and post 5.0 ± 4.4, *P* = 0.010	0.67
Dalrymple-Alford et al. (1995)^49^	Single-center, double-blind, placebo-controlled RCT, 8 weeks	21 patients, early PD not taking antiparkinsonian medication	1	65.7 (9.2)	1.7 (1.7)	BDI	No significant difference between groups; selegiline 10 mg, baseline 11.0 and post 7.0; placebo, baseline 10.0 and post 4.0, *P* value not shown	IC
Hietanen et al. (1991)^50^	Single-center, double-blind, placebo-controlled RCT, 4 weeks	20 patients, early PD not taking levodopa	1	56.9 (8.9)	4.2 (2.2)	BDI	No significant difference between groups; selegiline 30 mg, baseline 5 ± 4 and post 6 ± 3, placebo, baseline 6 ± 3 and post 5 ± 4, *P* value not shown	0.25
						HAMD	No significant difference between groups; selegiline 30 mg, baseline 5 ± 4 and post 4 ± 2, placebo, baseline 5 ± 3 and post 4 ± 3, *P* value not shown	0.25

Age and disease duration are presented as mean (SD) if available.

*BDI* Beck Depression Inventory, *FSS* Fatigue Severity Scale, *HADS* Hospital Anxiety and Depression Scale, *HAMD* Hamilton Depression Rating Scale, *HY stage* Hoehn–Yahr stage, *IC* incalculable, *NA* not assessed, *PD* Parkinson’s Disease, *PDQ* Parkinson’s Disease Questionnaire, *RCT* Randomized Controlled Trial.

**Table 3:** Sleep-related outcomes of MAO-BI studies.

Studies	Study design	Participants	Study quality	Age	Disease duration	Instruments	Outcome	Effect size
Biglan et al. (2006)^54^	Multicenter, double-blind, placebo-controlled RCT, 26 weeks	404 patients, early PD not requiring dopaminergic therapy	1	60.8 (10.8)	1.0 (1.2)	PDQUALIF: sleep	No significant difference between rasagiline 1 mg and placebo, −0.07, *P* = 0.69	IC
						PDQUALIF: sleep	No significant difference between rasagiline 2 mg and placebo, 0.02, *P* = 0.92	IC
Hauser et al. (2014)^28^	Multicenter, double-blind, placebo-controlled RCT, 18 weeks	321 patients, early PD not adequately controlled with dopamine agonizts	1	62.6 (9.7)	2.1 (2.1)	SCOPA daytime sleepiness	No significant differences between rasagiline 1 mg and placebo; statistics not shown	IC
Lim et al. (2015)^23^	Multicenter, double-blind, placebo-controlled RCT, 12 weeks	30 patients, PD with moderate to severe fatigue (FSS ≥ 4)	1	68.7 (7.4)	3 (median)	PDSS	No significant difference between rasagiline 1 mg and placebo (10.4 vs 3.25 points), *P* = 0.11	IC
Schrempf et al. (2018)^14^	Single-center, double-blind, placebo-controlled RCT, 8 weeks	20 patients, PD with sleep disturbances (PSQI > 5)	1	69.9 (6.9)	4.0 (3.5)	Polysomnography: sleep maintenance	Significant improvement with rasagiline 1 mg; baseline 62.1 ± 11.9 and post 70.6 ± 13.9, *p* = 0.024	0.71
						Polysomnography: sleep efficiency	No significant change with rasagiline 1 mg; baseline 58.1 ± 14.0 and post 63.5 ± 15.4, *p* = 0.097	0.39
						PDSS-2	No significant change with rasagiline 1 mg; baseline 19.6 ± 9.6 and post 20.1 ± 9.1, *p* = 0.798	0.04
						ESS	Significant improvement with rasagiline 1 mg; baseline 9.0 ± 4.8 and post 8.1 ± 4.7, *p* = 0.011	0.19
						PSQI	No significant change with rasagiline 1 mg; baseline 9.5 ± 2.6 and post 9.2 ± 2.5, p = 0.546	0.12
Panisset et al. (2016)^55^	Multicenter, open-label, prospective study, 2 months	110 PD patients not taking MAO-BI	3	67.0 (9.4)	3 (0–28)median (range)	PDSS	Significant improvement with rasagiline 0.5 or 1 mg; baseline 96.2 ± 21.6 and post 105.5 ± 21.9, *P* = 0.003	0.42
						ESS	No significant change with rasagiline 0.5 or 1 mg, baseline 10 ± 5.2 and post 9.4 ± 5.0, *p* = 0.4407	0.12
Schettino et al. (2016)^56^	Single-center, open-label, prospective study, 12 weeks	38 patients, mild-to-moderate PD with sleep disturbances (PDSS ≥ 100)	3	70.3 (10.6)	4.7 (0.5)	Patient sleep diaries: sleep latency time (h)	Significantly better in rasagiline + levodopa; rasagiline + levodopa −1.68 ± 1.21 vs. levodopa alone −0.55 ± 0.69, *P* = 0.001	IC
						Patient sleep diaries: total sleep time (h)	Significantly better in rasagiline + levodopa; rasagiline + levodopa 1.26 ± 1.62 vs. levodopa alone 0.32 ± 0.70, *P* = 0.026	IC
Müller et al. (2013)^15^	Single-center, open-label, prospective study, 4 months	30 patients, PD with sleep disturbances	3	66.6 (6.5)	NA	PDSS	Significantly improved after switching selegiline 7.5 mg to rasagiline 1 mg; baseline 111.3 ± 2.9 to 126.0 ± 2.0, *P* < 0.001	0.94
Liguori et al. (2018)^57^	Single-center, open-label, retrospective study, 4 months	15 patients, advanced PD with wearing off	4	70.0 (7.7)	6.2 (3.4)	PDSS-2	No significant change with rasagiline (dose not specified); baseline 19.5 ± 4.5 and post 17.8 ± 5.5, *P* value not shown	0.39
						PSQI	No significant change with rasagiline (dose not specified); baseline 7.3 ± 3.0 and post 6.5 ± 3.4, *P* value not shown	0.25
						ESS	No significant change with rasagiline (dose not specified); baseline 9.0 ± 2.1 and post 8.8 ± 3.7, *P* value not shown	0.09
Santos García et al. (2021)^25^	Multicenter, open-label, prospective study, 6 months	50 patients, PD with high non-motor burden (NMSS ≥ 40)	3	68.5 (9.1)	6.4 (5.1)	ESS	Significant improvement with safinamide 100 mg; baseline 9.2 ± 5.6 and post 6.9 ± 5.1, *P* = 0.012	0.40
						PSQI	Significant improvement with safinamide 100 mg; baseline 10.4 ± 4.0 and post 8.4 ± 4.4, *P* = 0.001	0.51
Liguori et al. (2018)^57^	Single-center, open-label, retrospective study, 4 months	46 patients, advanced PD with wearing off	4	70.0 (7.7)	6.2 (3.4)	PDSS-2	Significant improvement with safinamide (dose not specified); baseline 20.1 ± 12.1 and post 16.9 ± 10.6, *P* < 0.05	0.26
						PSQI	No significant change with safinamide (dose not specified); baseline 8.94 ± 4.38 and post 7.8 ± 3.6, *P* value not shown	0.27
						ESS	Significant improvement with safinamide (dose not specified); baseline 9.8 ± 5.5 and post 8.0 ± 4.5, *P* < 0.05	0.32
Plastino et al. (2021)^26^	Single-center, open-label, single-blinded, cross-over study, 12 weeks	30 patients, PD with RBD	3	65 (7.9)	6.0 (3.1)	PDSS-2	Significant improvement with safinamide 50 mg; baseline 20.0 ± 7.7 and post 17.3 ± 4.7, *P* = 0.042	0.35
						RBD questionnaire	Significant improvement with safinamide 50 mg; baseline 31.4 ± 12.4 and post 26.4 ± 12.4, *P* = 0.04	0.40
						ESS	No significant changes with safinamide 50 mg; statistics not shown	IC
						Polysomnography: total sleep time (min)	Significant improvement with safinamide 50 mg; baseline 400 ± 57 and post 427 ± 63, *P* = 0.041	0.47
Grigoriou et al. (2021)^43^	Multicenter, open-label, prospective study, 6 months	27 patients, advanced PD with off time > 1.5 h	3	65	6.8	PDSS-2	No significant change with safinamide 100 mg; baseline 14.8 ± 7.4 and post 13.8 ± 8.2, *p* = 0.35	0.14
De Micco et al. (2021)^44^	Single-center, open-label, prospective study, 6 months	20 patients, advanced PD with off time > 1.5 h	3	63.8 (10.2)	6.0 (2.2)	ESS	No significant change with safinamide 50 mg; baseline 5.50 ± 3.55 and post 4.20 ± 2.97, *P* = 0.42	0.24
						PDSS-2	No significant change with safinamide 50 mg; baseline 117.2 ± 21.4 and post 121.4 ± 17.7, *P* = 0.50	0.20
Bianchi et al. (2019)^45^	Single-center, open-label, retrospective study, 4.4 months	20 patients, advanced PD with motor fluctuations	4	75.0 (6.3)	14.5 (6.8)	PDSS-2	No significant change with safinamide 100 mg; baseline 122.4 ± 11.4 and post 125.6 ± 11.0, *P* = 0.44	0.28
Waters et al. (2004)^58^	Multicenter, double-blind, placebo-controlled RCT, 3 months	140 patients, advanced PD with off time > 3 h	1	65.3 (9.9)	6.7 (4.7)	Patient diaries: asleep time (h)	No significant differences between Zydis selegiline 1.25–2.5 mg and placebo; statistics not shown	IC
Gallazzi et al. (2021)^16^	Single-center, open-label, retrospective study, 3 months	45 patients, PD with excessive daytime sleepiness (ESS > 10 and/or PDSS item15 < 6)	4	65.4 (7.3)	6.8 (2.3)	ESS	Significant improvement with selegiline 10 mg; baseline 13.0 ± 4.2 and post 7.9 ± 4.3, *P* < 0.001	1.21

Age and disease duration are presented as mean (SD) if available.

*ESS* Epworth Sleepiness Scale, *FSS* Fatigue Severity Scale, *IC* incalculable, *NA* not assessed, *NMSS* Non-Motor Symptoms Scale, *PSQI* Pittsburgh Sleep Quality Index, *PD* Parkinson’s Disease; *PDSS* Parkinson’s Disease Sleep Scale, *RBD* REM sleep Behavior Disorder, *RCT* Randomized Controlled Trial.

**Table 4:** Pain outcomes of MAO-BI studies.

Studies	Study design	Participants	Study quality	Age	Disease duration	Instruments	Outcome	Effect size
Hattori et al. (2018)^31^	Multicenter, double-blind, placebo-controlled RCT, 26 weeks	404 patients, advanced PD with off time ≥ 2.5 hours	1	66.1 (8.3)	9.0 (4.7)	PDQ-39: bodily discomfort	Significantly better in rasagiline 1 mg vs placebo, −4.28 (−8.20 to −0.36), *p* = 0.0326	IC
						PDQ-39: bodily discomfort	No significant difference between rasagiline 0.5 mg and placebo, 1.00 (−4.85 to 2.85), *p* = 0.6099	IC
Zang et al. (2018)^32^	Multicenter, double-blind, placebo-controlled RCT, 16 weeks	324 patients, advanced PD with off time ≥ 1 hour	1	62.2 (9.4)	7.3 (4.6)	PDQ-39: bodily discomfort	Significantly better in rasagiline 1 mg vs placebo, −3.9 (−7.65 to −0.12), *p* = 0.043	IC
Hattori et al. (2019)^29^	Multicenter, double-blind, placebo-controlled RCT, 26 weeks	244 early PD patients not taking antiparkinsonian medication	1	66.4 (8.9)	1.8 (1.6)	PDQ-39: bodily discomfort	No significant differences between rasagiline 1 mg and placebo, −0.47 (−4.28 to 3.35), *P* = 0.8093	IC
Zhang et al. (2018)^30^	Multicenter, double-blind, placebo-controlled RCT, 26 weeks	130 early PD patients not taking antiparkinsonian medication	1	59.0 (8.9)	0.1 (median)	PDQ-39: bodily discomfort	No significant differences between groups; rasagiline 1 mg 2.14 ± 2.01 vs. placebo 1.28 ± 2.05, *P* = 0.749	IC
Barone et al. (2015)^17^	Multicenter, double-blind, placebo-controlled RCT, 12 weeks	123 patients, PD with moderate depression (BDI ≥ 15)	1	66.1 (8.5)	4.3 (12.5)	PDQ-39: bodily discomfort	No significant difference between groups, rasagiline 1 mg 2.01 ± 2.97 vs. placebo 2.72 ± 2.65, *P* value not shown	0.09
Hattori et al. (2019)^36^	Multicenter, open-label, prospective, phase 3 study, 52 weeks	222 PD patients taking levodopa with or without motor fluctuation	3	68.0 (8.4)	7.1 (5.0)	PDQ-39: bodily discomfort	No significant change with rasagiline 1 mg; baseline to post −1.29 ± 19.45, *P* value not shown	IC
Cibulcik et al. (2016)^21^	Single-center, open-label, prospective study, 3 months	42 patients, PD with freezing of gait	3	69.5 (7.9)	8.3 (4.3)	PDQ-39: bodily discomfort	Significant improvement with rasagiline 1 mg; baseline 27.5 ± 17.3 and post 23.4 ± 18.9, *p* = 0.039	0.24
Cattaneo et al. (2017)^108^	Post-hoc analysis of two multicenter, double-blind, placebo-controlled RCTs, 6 months	995 patients, advanced PD with off time > 1.5 h	1	60.9 (9.2)	8.6 (4.2)	PDQ-39 bodily discomfort	Significantly better in safinamide; safinamide 100 mg −5.28 ± 1.49 vs. placebo −1.59 ± 1.50, *P* = 0.0007	0.23
Borgohain et al. (2014)^37^	Multicenter, double-blind, placebo-controlled RCT, 24 weeks	669 patients, advanced PD with off time > 1.5 h	1	59.9 (9.4)	8.1 (3.9)	PDQ-39: bodily discomfort	Significantly better in safinamide; safinamide 100 mg −3.5 vs. placebo 0.2, *P* = 0.0159	0.16
						PDQ-39: bodily discomfort	No significant differences between groups; safinamide 50 mg −1.3 vs. placebo 0.2, *P* = 0.4937	0.06
Tsuboi et al. (2021)^59^	Multicenter, double-blind, placebo-controlled RCT, 24 weeks	406 patients, advanced PD with wearing off	1	68.1 (8.6)	8.2 (4.9)	PDQ-39: bodily discomfort	No significant differences between groups; safinamide 50 mg −1.71 ± 1.44 vs. placebo −2.94 ± 1.41, *P* = 0.5407	0.07
						PDQ-39: bodily discomfort	No significant differences between groups; safinamide 100 mg −6.13 ± 1.45 vs. placebo −2.94 ± 1.41, *P* = 0.118	0.28
Cattaneo et al. (2018)^60^	Post-hoc analysis of a multicenter, double-blind, placebo-controlled RCT, 2 years	355 patients, advanced PD with off time > 1.5 h	1	NA	NA	PDQ-39 bodily discomfort	Significantly better in safinamide 100 mg vs placebo, −3.66 (−6.71 to −0.60), *P* = 0.0190	IC
Santos García et al. (2021)^25^	Multicenter, open-label, prospective study, 6 months	50 patients, PD with high non-motor burden (NMSS ≥ 40)	3	68.5 (9.1)	6.4 (5.1)	King’s PD pain scale	Significant improvement with safinamide 100 mg; baseline 40.0 ± 36.2 and post 22.6 ± 21.4, *P* < 0.0001	0.48
						Visual Analog Scale: pain	No significant change with safinamide 100 mg; baseline 4.6 ± 3.2 and post 3.7 ± 2.7, *P* = 0.071	0.29
						PDQ-39: bodily discomfort	Significant improvement with safinamide 100 mg; baseline 44.6 ± 27.4 and post 33.3 ± 19.9, *P* = 0.018	0.41
Grigoriou et al. (2021)^43^	Multicenter, open-label, prospective study, 6 months	27 patients, advanced PD with off time > 1.5 ho	3	65	6.8	King’s PD pain scale	Significant improvement with safinamide 100 mg; mean score, baseline 18.0 and post 12.4, *P* = 0.02	IC
De Micco et al. (2021)^44^	Single-center, open-label, prospective study, 6 months	20 patients, advanced PD with off time > 1.5 h	3	63.8 (10.2)	6.0 (2.2)	King’s PD Pain Scale	No significant change with safinamide 50 mg; baseline 9.40 ± 7.88 and post 8.60 ± 9.20, *P* = 0.77	0.10
Geroin et al. (2020)^46^	Single-center, open-label, prospective study, 12 weeks	13 patients, advanced PD with motor fluctuation and pain (NRS ≥ 4)	3	64.1 (6.7)	5.8 (2.9)	King’s PD pain scale	Significant improvement with safinamide 100 mg; baseline to post −19.3 ± 10.5, *P* < 0.05	IC
						Brief Pain Inventory: Intensity	Significant improvement with safinamide 100 mg; baseline to post −11.8 ± 5.2, *P* < 0.05	IC
						Brief Pain Inventory: Interference	Significant improvement with safinamide 100 mg; baseline to post −24.4 ± 11.1, *P* < 0.05	IC
						NRS	Significant improvement with safinamide 100 mg; baseline to post −4.6 ± 1.9, *P* < 0.05	IC
						PDQ-39 bodily discomfort	Significant improvement with safinamide 100 mg; baseline to post −4.5 ± 2.4, *P* < 0.05	IC

Age and disease duration are presented as mean (SD) if available.

*BDI* Beck Depression Inventory, *IC* incalculable, *ICD* Impulse Control Disorders, *NA* not assessed, *NMSS* Non-Motor Symptoms Scale, *NRS* Numeric Rating Scale, *PD* Parkinson’s Disease, *PDQ* Parkinson’s Disease Questionnaire, *RCT* Randomized Controlled Trial.

**Table 5:** Fatigue outcomes of MAO-BI studies.

Studies	Study design	Participants	Study quality	Age	Disease duration	Instruments	Outcome	Effect size
Stocchi et al. (2014)^62^	Multicenter, double-blind, placebo-controlled RCT, 36 weeks	1105 patients, early PD not requiring dopaminergic therapy	1	62.2 (9.7)	4.5 (4.6)	Parkinson’s fatigue scale	Significantly better in rasagiline 1 mg vs placebo, −0.14 ± 0.05, *P* < 0.01	0.03
						Parkinson’s fatigue scale	Significantly better in rasagiline 2 mg vs placebo, −0.19 ± 0.05, *P* < 0.0001	0.02
Lim et al. (2015)^23^	Multicenter, double-blind, placebo-controlled RCT, 12 weeks	30 patients, PD with moderate to severe fatigue (FSS ≥ 4)	1	68.7 (7.4)	3 (median)	Modified fatigue impact Scale	Significantly better in rasagiline 1 mg vs placebo (12 vs 8.5 points), *P* = 0.003	IC
					3 (median)	FSS	Significantly better in rasagiline 1 mg vs placebo (13 vs 3 points), *P* = 0.027	IC
					3 (median)	Multidimensional fatigue inventory	Significantly better in rasagiline 1 mg vs placebo (5 vs 1 points), *P* = 0.04	IC
					3 (median)	Objective physical and mental fatigue testing	No significant differences between rasagiline 1 mg and placebo (0 vs 0.07 points), *P* = 0.26	IC
Santos García et al. (2021)^25^	Multicenter, open-label, prospective study, 6 months	50 patients, PD with high non-motor burden (NMSS ≥ 40)	3	68.5 (9.1)	6.4 (5.1)	Visual analog fatigue scale: physical	No significant change with safinamide 100 mg; baseline 4.2 ± 2.8 and post 3.6 ± 2.6, *P* = 0.293	0.19
						Visual analog fatigue scale: mental	No significant change with safinamide 100 mg; baseline 3.1 ± 2.7 and post 2.5 ± 2.8, *P* = 0.118	0.26
De Micco et al. (2021)^44^	Single-center, open-label, prospective study, 6 months	20 patients, advanced PD with off time > 1.5 h	3	63.8 (10.2)	6.0 (2.2)	PD fatigue scale	Significant improvement with safinamide 50 mg; baseline 2.85 ± 0.67 and post 2.20 ± 1.07, *P* = 0.02	0.97
Bianchi et al. (2019)^45^	Single-center, open-label, retrospective study, 4.4 months	20 patients, advanced PD with motor fluctuations	4	75.0 (6.3)	14.5 (6.8)	Physical fatigue scales	No significant change with safinamide 100 mg; baseline 39.4 ± 19.8 and post 39.4 ± 22.5, *P* = 1.00	0.00
						Mental fatigue scales	No significant change with safinamide 100 mg; baseline 20.0 ± 17.0 and post 20.0 ± 14.1, *P* = 1.00	0.00

Age and disease duration are presented as mean (SD) if available.

*FSS* Fatigue Severity Scale, *IC* incalculable, *NMSS* Non-Motor Symptoms Scale, *PD* Parkinson’s Disease, *RCT* Randomized Controlled Trial.

**Table 6:** Autonomic function outcomes of MAO-BI studies.

Studies	Study design	Participants	Study quality	Age	Disease duration	Instruments	Outcome	Effect size
Biglan et al. (2006)^54^	Multicenter, double-blind, placebo-controlled RCT, 26 weeks	404 patients, early PD not requiring dopaminergic therapy	1	60.8 (10.8)	1.0 (1.2)	PDQUALIF: urinary function	No significant difference between rasagiline 1 mg and placebo, 0.14, *P* = 0.39	IC
						PDQUALIF: urinary function	No significant difference between rasagiline 2 mg and placebo, 0.00, *P* = 0.99	IC
Brusa et al. (2014)^63^	Single-center, open-label, prospective study, 2 months	20 patients, early PD patients with HY stage ≤ 2.5	3	67 (3.2)	5.0 (2.1)	Urodynamics: first sensation (ml)	Significant improvement with rasagiline 1 mg; baseline 118 ± 53 and post 158 ± 42, *p* < 0.001	0.75
						Urodynamics: bladder capacity (ml)	No significant change with rasagiline 1 mg; baseline 170 ± 86 and post 188 ± 73, NS	0.21
						Urodynamics: First sensation (ml)	Significant improvement with rasagiline 1 mg; baseline 290 ± 98 and post 337 ± 115, *p* < 0.001	0.48
						Urodynamics: residual urine (ml)	Significant improvement with rasagiline 1 mg; baseline 47 ± 23 and post 25 ± 15, *p* < 0.001	0.96
						International Prostate Symptoms Score questionnaire	Significant improvement with rasagiline 1 mg; baseline 12.3 ± 2.1 and post not shown, *p* < 0.0005	IC
Gómez-López et al. (2021)^24^	Single-center, open-label, retrospective study, 3 months	114 patients, PD with urinary symptoms	4	72.6 (10.0)	6.9 (6.1)	SCOPA-AUT: urinary problems	Significant improvement with safinamide 100 mg; baseline 9.1 ± 3.1 and post 6.6 ± 3.0, *P* < 0.0001	0.81
Santos García et al. (2021)^25^	Multicenter, open-label, prospective study, 6 months	50 patients, PD with high non-motor burden (NMSS ≥ 40)	3	68.5 (9.1)	6.4 (5.1)	NMSS: urinary symptoms	Significant improvement with safinamide 100 mg; baseline 42.72 ± 30.41 and post 30.62 ± 23.94, *p* = 0.003	0.40
						NMSS: cardiovascular	No significant change with safinamide 100 mg; baseline 9.58 ± 2.46 and post 6.72 ± 11.94, *p* = 0.268	1.16
						NMSS: gastrointestinal symptoms	Significant improvement with safinamide 100 mg; baseline 19.61 ± 18.01 and post 13.13 ± 13.39, *p* = 0.01	0.36
						NMSS: sexual dysfunction	No significant change with safinamide 100 mg; baseline 28.25 ± 35.69 and post 25.28 ± 33.58, *p* = 0.784	0.08
De Micco et al. (2021)^44^	Single-center, open-label, prospective study, 6 months	20 patients, advanced PD with off time > 1.5 h	3	63.8 (10.2)	6.0 (2.2)	SCOPA-AUT	Significant improvement with safinamide 50 mg; baseline 12.8 ± 5.69 and post 7.95 ± 4.40, *P* = 0.04	0.85

Age and disease duration are presented as mean (SD) if available.

*HY stage* Hoehn–Yahr stage, *IC* incalculable, *NMSS* Non-Motor Symptoms Scale, *PD* Parkinson’s Disease, *RCT* Randomized Controlled Trial, *SCOPA* SCales for Outcomes in PArkinson’s disease.

**Table 7:** Apathy, olfaction, and ICD outcomes of MAO-BI studies.

Studies	Study design	Participants	Study quality	Age	Disease duration	Instruments	Outcome	Effect size
Barone et al. (2015)^17^	Multicenter, double-blind, placebo-controlled RCT, 12 weeks	123 patients, PD with moderate depression (BDI ≥ 15)	1	66.1 (8.5)	4.3 (12.5)	Apathy scale	No significant difference between rasagiline 1 mg and placebo, statistics not shown	IC
Lim et al. (2015)^23^	Multicenter, double-blind, placebo-controlled RCT, 12 weeks	30 patients, PD with moderate to severe fatigue (FSS ≥ 4)	1	68.7 (7.4)	3 (median)	Marin Apathy inventory	No significant differences between rasagiline 1 mg and placebo (2 vs 0.5 points), *P* = 0.32	IC
De Micco et al. (2021)^44^	Single-center, open-label, prospective study, 6 months	20 patients, advanced PD with off time > 1.5 h	3	63.8 (10.2)	6.0 (2.2)	Apathy evaluation scale	Significant improvement with safinamide 50 mg; baseline 34.65 ± 7.41 and post 30.35 ± 7.80, *P* = 0.01	0.58
Hauser et al. (2014)^28^	Multicenter, double-blind, placebo-controlled RCT, 18 weeks	321 patients, early PD not adequately controlled with dopamine agonizts	1	62.6 (9.7)	2.1 (2.1)	Brief smell identification test	No significant differences between groups; rasagiline 1 mg −0.1 ± 2.2 vs. placebo −0.0 ± 1.9, *P* value not shown	IC
Haehner et al. (2013)^69^	Single-center, double-blind, placebo-controlled RCT, 120 days	34 patients with PD	1	59.1 (9.0)	2.9 (1.8)	Sniffin’ Sticks test kit	No significant differences in threshold, discrimination, and identification were found between rasagiline 1 mg and placebo (all *P* > 0.05)	IC
						Olfactory event related potential	3-factorial ANOVA showed no significant main effects of drug (rasagiline vs placebo), session (baseline vs 120 days), or stimulant (all *P* > 0.05)	IC
De Micco et al. (2021)^44^	Single-center, open-label, prospective study, 6 months	20 patients, advanced PD with off time > 1.5 h	3	63.8 (10.2)	6.0 (2.2)	Questionnaire for ICD in PD rating scale	No significant change with safinamide 50 mg; baseline 0.75 ± 2.04 and post 1.15 ± 2.18, *P* = 0.55	0.20

Age and disease duration are presented as mean (SD) if available.

*BDI* Beck Depression Inventory, *IC* incalculable, *ICD* Impulse Control Disorders, *PD* Parkinson’s Disease, *RCT* Randomized Controlled Trial.

### Quality of life (Table 1)

The Parkinson’s Disease Questionnaire-39 (PDQ-39) was most commonly used to estimate the changes in QOL after MAO-BI administration^27^. Eight double-blind, placebo-controlled RCTs reported the effects of rasagiline on QOL based on PDQ-39: three RCTs for patients with early PD^28–30^, two for those with advanced PD^31,32^, one for those with moderate depression^17^, one for those with moderate to severe fatigue^23^, and one for those with sleep disturbances^14^. Of these studies, only two studies reported significant benefits of rasagiline on QOL at 12–26 weeks (one RCT on advanced PD patients and another RCT for those with moderate to severe fatigue)^23,31^. Based on the results from the RCTs, effect sizes for rasagiline 1 mg and 0.5 mg were trivial to small (0.05–0.27) and trivial (0.03), respectively. Two Chinese RCTs (one for early PD and one for advanced PD) found significant benefits of rasagiline on QOL based on the EuroQol 5 dimensions (EQ-5D) despite the non-significant results based on the PDQ-39^30,32^. Two old RCTs evaluated QOL outcomes based on the PDQUALIF scale^33,34^; significant improvement in the PDQUALIF scale was observed not in early PD patients but in advanced PD patients. In addition, open-label studies reported significant^35^ or non-significant^21,36^ benefits of rasagiline based on the PDQ-39.

All the studies of safinamide enrolled advanced PD patients^37–46^ except for one study for those with high non-motor burden (defined as the NMS Scale (NMSS) ≥ 40)^25^. RCTs and open-label studies using safinamide 100 mg reported positive or negative QOL outcomes with safinamide^25,37–40,42,43,45,46^, whereas all the studies using safinamide 50 mg reported negative outcomes^37,39,40,44^. Based on the PDQ-39 outcomes from the RCTs, effect sizes for safinamide 100 mg and 50 mg were small (0.22–0.23) and trivial (0.11–0.15), respectively.

Collectively, a minority of the RCTs (rasagiline or safinamide vs. placebo) for advanced PD patients reported statistically significant QOL improvement. There have been no selegiline studies reporting QOL outcomes. The clinical impact of the QOL changes will be discussed later.

### Depression and anxiety (Table 2)

The ACCORDO study, a multicenter, double-blind, placebo-controlled RCT, enrolled non-demented PD patients with moderately severe depressive symptoms (Beck Depression Inventory, BDI ≥ 15)^17^. Compared with placebo, rasagiline 1 mg led to a significantly larger reduction in the BDI scores at 4 weeks (effect size, 1.01) without a significant between-group difference at 12 weeks. Another multicenter, double-blind, placebo-controlled RCT enrolled 30 PD patients with moderate to severe fatigue^23^. In parallel with fatigue improvement, rasagiline 1 mg showed a significantly greater reduction of the BDI scores than placebo (5.5 vs. 0.5 points, *P* = 0.018). In contrast, significant improvement in anxiety was not observed based on the State-Trait Anxiety Inventory. Significant benefits of rasagiline on depressive symptoms over placebo were not observed in RCTs either for early PD patients or those with MCI^19,34,47^. Seven studies assessed the impact on anxiety and depressive symptoms according to the PDQ-39 emotional well-being subscore^17,21,29–32,36^; a minority of studies for either early or advanced PD patients reported significant benefits of rasagiline^29,31^.

In a multicenter, double-blind, placebo-controlled RCT^38^ and two open-label prospective studies^43,44^, safinamide 100 mg for advanced PD patients did not significantly ameliorate depression at 6 months. However, in the Study 016 and its extension study (Study 018), significant benefits on the Hamilton Depression Scale (HAMD) and PDQ-39 emotional well-being subscore over placebo were observed with safinamide 100 mg but not with the 50 mg dose^37,39^. Furthermore, a post hoc analysis of these two studies confirmed the beneficial effects of safinamide 100 mg on depression^48^. Interestingly, compared with the placebo group, significantly fewer patients in the safinamide group experienced depression as adverse events during the 2-year follow-up^48^. Also, open-label studies on patients with either depressive symptoms^18^ or high non-motor burden^25^ reported prominent improvement in depression with safinamide based on the HAMD or BDI-II, respectively. The Study 016, its post hoc analysis, and an open-label study observed significant improvement in the PDQ-39 emotional well-being subscore^37,48^. However, none of the two open-label prospective studies found significant benefits of safinamide on anxiety^43–45^.

Five double-blind, placebo-controlled RCTs for early PD patients reported the effects of selegiline on depressive symptoms using either the HAMD or BDI. Two small RCTs reported negative results^49,50^, whereas three large RCTs suggested a positive impact of selegiline 10 mg on depressive symptoms with trivial to moderate effect sizes (0.10 and 0.67)^51–53^.

Taken together, rasagiline, safinamide, and selegiline may potentially improve depressive symptoms. In contrast, no studies demonstrated significant benefits of MAO-BIs on anxiety.

### Sleep disturbances (Table 3)

Two large multicenter, double-blind, placebo-controlled RCTs for early PD patients reported non-significant effects of rasagiline 1 mg on sleep disturbances at 18–26 weeks based on the PDQUALIF sleep subscore or Scales for Outcomes in PD (SCOPA) daytime sleepiness score^28,54^. Similarly, one small multicenter, double-blind, placebo-controlled RCT for PD patients with moderate to severe fatigue demonstrated non-significant effects of rasagiline 1 mg on sleep disturbances based on the Parkinson’s Disease Sleep Scale (PDSS) at 12 weeks^23^. One single-center, double-blind, placebo-controlled RCT enrolled 20 PD patients with sleep disturbances^14^. Rasagiline 1 mg led to significantly better sleep maintenance as assessed by polysomnography (effect size, 0.71), with significantly decreased wake time after sleep onset, number of arousals, and percentage of light sleep. Although daytime sleepiness, as measured by the Epworth Sleepiness Scale (ESS), improved significantly with rasagiline (effect size, 0.19), there was no significant change in the PDSS-2. The authors found no correlations of polysomnographic sleep parameters or PDSS-2 score with changes in motor function^14^. Open-label studies reported positive^15,55,56^ or non-significant^57^ effects of rasagiline on sleep disturbances.

Six open-label studies have reported the impact of safinamide on sleep disturbances^25,26,43–45,57^. Most studies employed the PDSS-2 and ESS; however, the outcomes were inconsistent. An open-label cross-over study enrolled 30 PD patients with RBD^26^. Interestingly, safinamide 50 mg alleviated RBD as assessed by polysomnography and questionnaires at 3 months. This study is the only one investigating the effects of MAO-BIs on RBD.

In a large multicenter, double-blind, placebo-controlled RCT for advanced PD patients, orally disintegrating selegiline 1.25–2.5 mg did not prolong asleep time based on patient diaries^58^. A single-center, open-label, retrospective study analyzed the effects of selegiline 10 mg on excessive daytime sleepiness at 3 months^16^. The authors reported significant alleviation of excessive daytime sleepiness based on the ESS with a large effect size (1.21). This benefit was accompanied by improved self-perceived quality of sleep. In a single-center, open-label, prospective study, switching from selegiline 7.5 mg to rasagiline 1 mg led to an improvement in sleep disturbances based on the PDSS^15^. However, the results should be cautiously interpreted because of the potential effect of patients’ expectations of treatment benefits.

In summary, no RCTs showed significant benefits of MAO-BIs based on the sleep-specific rating scales. However, one RCT using polysomnography and some open-label studies reported positive effects of MAO-BIs on sleep disturbances.

### Pain (Table 4)

All the rasagiline studies (6 large RCTs and 1 small open-label study) reported pain outcomes based on the PDQ-39 bodily discomfort subscore^17,21,29–32,36^. The two large RCTs for advanced PD patients showed significant benefits of rasagiline 1 mg on pain at 16–26 weeks (effect size, incalculable)^31,32^. Alleviation of pain with rasagiline 0.5 mg was numerically smaller and did not reach statistical significance^31^. Two large RCTs for early PD patients did not find significant benefits of rasagiline 1 mg on pain^29,30^.

In a large double-blind, placebo-controlled RCT, advanced PD patients were randomized to safinamide 100 mg, 50 mg, or placebo for 24 weeks^37^. Compared with placebo, only patients taking safinamide 100 mg experienced significant amelioration of pain based on the PDQ-39 bodily discomfort subscore. Similar results were observed in another large RCT for advanced PD patients, although the outcomes did not reach statistical significance^59^. Furthermore, small open-label studies and post hoc analyses of large RCTs for advanced PD patients support the efficacy of safinamide 100 mg on pain^25,46,60^. Two open-label studies reported detailed outcomes based on the King’s PD pain scale, suggesting that safinamide 100 mg improves fluctuation-related pain^43,61^. Collectively, safinamide 100 mg possibly ameliorates pain, especially fluctuation-related pain, with trivial to small effect size (0.16–0.41).

Collectively, rasagiline and safinamide might improve pain, especially in patients with more advanced disease stages. Note that no selegiline studies have reported pain outcomes.

### Fatigue (Table 5)

A large multicenter, double-blind, placebo-controlled RCT for early PD patients reported a significant difference in fatigue favoring rasagiline compared with placebo at 36 weeks^62^. However, the effect sizes were trivial (0.03 and 0.02 for rasagiline 1 and 2 mg, respectively). In a small multicenter, double-blind, placebo-controlled RCT for PD patients with moderate to severe fatigue, fatigue significantly improved with rasagiline 1 mg at 12 weeks (effect size, incalculable)^23^.

Three open-label prospective studies of safinamide reported contrasting findings. One study showed significant alleviation of fatigue with a large effect size (0.97)^44^, while the other two reported non-significant results^25,45^.

Taken together, the number of studies reporting fatigue outcomes remains scarce, and the impact of MAO-BIs on fatigue appears inconsistent across studies.

### Autonomic dysfunctions (Table 6)

A large RCT for early PD patients found no significant benefits of rasagiline on urinary symptoms based on the PDQUALIF urinary function subscore at 26 weeks^54^. In a small single-center, open-label, prospective study, urodynamic evaluations revealed a significant gain in volume variables after the 2-month administration of rasagiline 1 mg^63^.

In a small single-center, open-label, prospective study for advanced PD patients, safinamide 50 mg ameliorated overall autonomic symptoms based on the SCOPA-Autonomic at 6 months^44^. The result for each domain was not reported. A large single-center, open-label, retrospective study showed that safinamide 100 mg reduced the SCOPA-Autonomic urinary problems subscore at 3 months^24^. The benefits were driven mainly by alleviating incontinence, urgency, daily frequency, and nocturia. A multicenter, open-label, prospective study on PD patients with high non-motor burden reported the effects of safinamide 100 mg on autonomic symptoms using the NMSS subscore^25^. Significant improvement was observed not in cardiovascular and sexual symptoms domains but in gastrointestinal and urinary symptoms domains.

In summary, the effects of MAO-BIs on various autonomic symptoms remain unclear because of the scarcity of data.

### Cognitive dysfunctions

A total of 29 studies tested the effects of MAO-BIs on cognitive functions using various assessment batteries (Supplementary Table 2). No studies for either early or advanced PD patients found significant benefits in global cognition based on the Mini Mental State Examination (MMSE), Montreal Cognitive Assessment (MoCA), or SCOPA-Cognition^20,22,28,38,44,45,52,64–66^. Likewise, except for the following ones, most studies did not find beneficial effects of MAO-BIs using domain-specific cognition assessment batteries.

Two multicenter, double-blind, placebo-controlled RCTs investigated the effects of rasagiline 1 mg on cognitive functions in PD patients with MCI^19,20^. One RCT assessed the effects of rasagiline on global cognition and cognition-related instrumental activities of daily living based on the SCOPA-Cognition, MoCA, and Penn Daily Activities Questionnaire but failed to show significant benefits^20^. The other RCT reported the effects of rasagiline on various cognitive domains: attention, executive functions, memory, visuospatial functions, and language^19^. Although digit span–backward and verbal fluency total scores showed significantly better outcomes in rasagiline compared with placebo, the additional analysis showed significant benefits of rasagiline only in the attentional domain.

Two single-center open-label prospective studies from the same group investigated the effects of MAO-BIs in PD patients with wearing-off^67,68^. The unique point of these studies was that cognitive assessments were performed 20 min before the second scheduled daily dose of levodopa. Consequently, executive functions improved with either rasagiline or safinamide.

Collectively, MAO-BIs are unlikely to improve global cognition but might have the potential to improve fluctuation-related cognitive dysfunctions.

### Miscellaneous: apathy, olfactory dysfunctions, and ICD (Table 7)

In a small single-center, open-label, prospective study for advanced PD patients, significant improvement in apathy was observed 6 months after administrating safinamide 50 mg with a moderate effect size (0.58)^44^. Conversely, multicenter RCTs did not find significant benefits of rasagiline on apathy^17,23^. Two double-blind, placebo-controlled RCTs tested the effects of rasagiline 1 mg on olfactory functions with non-significant benefits^28,69^. A single-center, open-label, prospective study reported no significant impact of safinamide 50 mg on ICD based on the Questionnaire for ICD in PD rating scale (QUIP-RS)^44^.

## Discussion

This systematic review summarized the QOL and NMS outcomes drawn from the available clinical studies of MAO-BIs. The impact of MAO-BIs on QOL was inconsistent across studies, and this was unlikely to be clinically meaningful. Overall, rasagiline and safinamide had more evidence supporting improvements in NMS when compared with selegiline. MAO-BIs potentially improve depression, sleep disturbances, and pain (particularly pain related to motor fluctuations). In contrast, MAO-BIs are unlikely to improve cognitive and olfactory dysfunctions. Given the paucity of evidence, the effects of MAO-BIs on fatigue, autonomic dysfunctions, apathy, and ICD remain unknown. As the recent review on NMS treatment by the Movement Disorders Society demonstrated, there still remain significant unmet needs in this field^5^. Thus, the potential roles of MAO-BIs in the treatment of NMS will be discussed in the following paragraphs.

A subset of RCTs for advanced PD patients demonstrated statistically significant benefits of rasagiline or safinamide on QOL, with trivial to small effect sizes^23,31,37–39,41^. The minimal clinically important difference (MCID) is the smallest difference in scores that are subjectively meaningful to patients. The MCID threshold for the PDQ-39 summary index was reported to be −4.72 (improvement) and +4.22 (worsening)^70^. No MAO-BI studies demonstrated improvement of the PDQ-39 summary index larger than this MCID threshold. Thus, the impact of MAO-BI on overall QOL may not be clinically meaningful.

Depression and anxiety are among the most common NMS in PD and are key determinants of QOL^71^. Past studies showed beneficial effects of rasagiline 1 mg^23^, safinamide 100 mg^37,39,48^, and selegiline 10 mg^51–53^ for depressive symptoms, although the results were inconsistent across studies. Of note, most RCTs of MAO-BIs excluded patients with clinically-relevant depression or patients on concurrent antidepressants for safety reasons^19,34,37–39,47–49,51–53,64,65^, possibly in some studies accounting for non-significant results. A multicenter, double-blind, placebo-controlled RCT (ACCORDO) evaluated the effects of rasagiline 1 mg on depressive symptoms in depressive PD patients^17^. The primary efficacy variable, the BDI scores, improved significantly in the rasagiline group compared with placebo at 4 weeks (effect size, 1.01), without a significant between-group difference at 12 weeks. In the afore-mentioned review on NMS treatment by the Movement Disorders Society, pramipexole was the only parkinsonian medication, classified as “efficacious” for depressive symptoms^5^. This was mainly based on the positive results from a multicenter, double-blind, placebo-controlled RCT for PD patients with depressive symptoms, where the BDI scores decreased significantly in the pramipexole group compared with placebo at 12 weeks (−5.9 ± 0.5 vs. −4.0 ± 0.5, *P* = 0.01)^72^. The results from the ACCORDO and the above-mentioned pramipexole study seem comparable. The negative results for the ACCORDO study might be partly explained by large placebo effects at 12 weeks (rasagiline 1 mg −5.40 ± 0.79 vs. placebo −4.43 ± 0.73, *P* = 0.368), as patients with depression are known to show high response rates to placebo^73^. Another issue will be the safety of co-administrating MAO-BIs and antidepressants. A few studies for large PD cohorts did not observe serotonin syndrome despite the combined therapy^18,74,75^. Although serotonin syndrome seems rare, the long-term safety of the combined therapy needs to be clarified because of the potentially fatal nature of the serotonin syndrome. Finally, three studies using either rasagiline or safinamide assessed anxiety, and all three had negative results^19,22,23^. The positive effects of MAO-BIs on anxiety, therefore, remain unproven.

Nocturnal sleep disturbances include difficulty initiating sleep, difficulty maintaining sleep, and early morning awakenings; another issue for PD patients is excessive daytime sleepiness^76^. A small placebo-controlled RCT for PD patients with sleep disturbances demonstrated, using polysomnography, significantly better sleep maintenance with rasagiline 1 mg with statistically non-significant improvement in sleep efficacy by 9.3%^14^. However, other studies of rasagiline, safinamide, or selegiline reported contrasting findings presumably because sleep disturbances in PD patients are multifactorial: e.g., nocturnal hypokinesia, nocturia, pain, muscle cramps, restless legs syndrome, RBD, or adverse effects of medications^76^. Therefore, treatment options should be tailored to the putative etiology of patients’ sleep disturbances. In the review on NMS treatment by the Movement Disorders Society^5^, no treatment options were classified as “efficacious” for sleep disturbances, and rotigotine was the only parkinsonian medication that was classified as “likely efficacious.” A small placebo-controlled RCT assessed the impact of rotigotine on nocturnal sleep using polysomnography in advanced PD patients with sleep disturbances^77^. Consequently, rotigotine administration led to significantly larger improvement in sleep efficacy as compared with placebo (8.0% vs. 0.5%, *P* < 0.001). In other studies, ropinirole and rotigotine have been shown to improve nocturnal sleep disturbances mainly by improving nocturnal motor symptoms^78–80^. MAO-BIs potentially improve sleep disturbances through a similar mechanism; however, this remains speculative.

Multiple etiologies of pain in PD patients have been suggested: fluctuation-related, central, musculoskeletal, or neuropathic pain^81^. However, in the review on NMS treatment by the Movement Disorders Society^5^, no treatment options were labeled as “efficacious” or “likely efficacious” for pain. Rasagiline and safinamide have been beneficial for pain in advanced PD patients^31,32,37^. Detailed investigations on pain, based on the King’s PD pain scale, have suggested that fluctuation-related pain responded best to safinamide 100 mg^43,61^. Similarly, dopamine agonizts such as ropinirole or apomorphine have been reported to improve fluctuation-related pain^82,83^. Therefore, compared with patients with early PD, those with advanced PD might benefit more from long-acting dopaminergic agents (e.g., MAO-BIs or dopamine agonizts) in pain relief.

Accumulating evidence suggests that MAO-BIs are unlikely to improve cognitive and olfactory dysfunctions^20,22,28,38,44,45,52,64–66,69^. The limited available evidence did not allow us to determine the effects of MAO-BIs on fatigue, autonomic dysfunctions, apathy, and ICD. Note that selegiline suppressed cardiovascular autonomic responses^84,85^ and could result in orthostatic hypotension^86–88^. Clinicians should be aware of this potential adverse effect, as it may increase the risk of falling^89^. The pathophysiology of NMS remains uncertain but may involve both dopaminergic and non-dopaminergic dysfunctions^11^. Further clinical and preclinical investigations will be required.

There are several possible mechanisms for MAO-BIs improving NMS. MAO-BIs may improve nocturnal sleep disturbances or pain by improving motor symptoms and motor fluctuations^90^. Other NMS might improve through alleviation of non-motor fluctuations^91^. Open-label studies have suggested that executive dysfunctions related to non-motor fluctuations improved with MAO-BIs^67,68^. Future studies should investigate the effects of MAO-BIs specifically on NMS with or without fluctuations. The Non-Motor Fluctuation Assessment Questionnaire (NoMoFA) was recently validated and should be helpful to capture static and fluctuating NMS^92^. In addition, the effects of MAO-BIs on MAO-A, which metabolizes catecholamines and serotonin, might account for part of the effects of MAO-BIs^1^.

Another potential mechanism of action exclusively for safinamide is the modulation of overactive glutamatergic tone^2,3^. Increasing evidence from preclinical and clinical studies supports the importance of glutamatergic transmission in motor and NMS of PD^93^. Since safinamide 50 mg completely inhibits MAO-B activity, additional benefits with safinamide 100 mg might be due to non-dopaminergic mechanisms^1^. Interestingly, post hoc analysis of a large placebo-controlled RCT reported that safinamide 100 mg might improve dyskinesia^94^. This effect was analogous to the dyskinesia-suppressing effects of amantadine, an NMDA glutamate receptor antagonist^95^. In addition, a large RCT demonstrated that safinamide 100 mg improved depressive symptoms and pain with greater effect sizes compared with safinamide given at 50 mg^39^. The clinical relevance of dopaminergic and non-dopaminergic effects of safinamide needs further exploration.

We would like to highlight the limitations of the current study. First, most studies included were RCTs reporting QOL or non-motor results as the secondary outcomes or open-label studies. Therefore, the outcomes should be cautiously interpreted. Second, considerable inconsistency exists among studies. The variation in the inclusion criteria, assessment batteries, follow-up periods, and numbers of participants may account for the highly variable outcomes. Note that QOL or NMS assessment batteries used in some studies were not recommended for PD patients^96–101^. Genetic diversity may also have an effect on the variability of outcomes, as polymorphisms of MAO-A, MAO-B, and catechol-o-methyltransferase (COMT) have been associated with the occurrence of motor fluctuations in PD patients and psychiatric symptoms in non-PD populations^102–104^. Third, as most studies reported relatively short-term outcomes, longer-term data are warranted. Finally, another important thing to discuss is how to determine the impact of the various interventions. MCID avoids the issue of mere statistical significance and provides a threshold at which results are clinically meaningful. To the best of our knowledge, MCID for non-motor assessment batteries has not been studied in PD patients, except for the PDSS-2^105^. Alternatively, we calculated effect sizes to estimate the clinical relevance of interventions despite criticism about this method^106^. Unfortunately, required data were missing in some studies. Standardized reporting of the results, including numbers of patients and, mean scores with SD at baseline and post-intervention, will be essential as these data are also needed for future meta-analyses.

In conclusion, MAO-BIs may potentially improve depression, sleep disturbances, and pain. In contrast, MAO-BI administration may not lead to clinically-meaningful improvement in QOL or cognitive and olfactory dysfunctions. The effects of MAO-BIs on other NMS remain unclear. If NMS is related to poor motor symptoms, or if NMS fluctuates along with blood levodopa concentration, the NMS may be more likely to improve with MAO-BIs. With the increasing number of treatment options available, it will be important to compare the efficacy of MAO-BIs with other options, such as dopamine agonizts and COMT inhibitors. Especially, the effects of these agents on static and fluctuating NMS should be investigated in future studies. Non-dopaminergic effects of safinamide are also of great interest. Ideally, clinicians should be able to provide personalized medicine based on patients’ symptoms and genetic profiles. By drawing attention to the gaps in knowledge, we hope to encourage researchers to conduct high-quality research exploring the efficacy of MAO-BIs and other agents on NMS for persons living with PD.

## Methods

### Search strategy

We conducted a systematic literature search from January 1990 to November 2021 using the PubMed, Scopus, and Cochrane Library databases according to the Preferred Reporting Items for Systematic Reviews and Meta-analysis (PRISMA) guidelines. The search terms included Parkinson’s disease, Parkinson disease, selegiline, rasagiline, and safinamide. The search syntax is provided in Supplementary Material. Two investigators (TT and YS) independently screened all records for duplicates and then performed title/abstract screening and full-text assessments based on the eligibility criteria below. Disagreements were resolved through review of the primary study and expert discussion.

### Eligibility criteria and calculation of effect sizes

The inclusion criteria for this systematic review were: (1) clinical studies on patients with Parkinson’s disease (*n* ≥ 10), (2) reporting the effects of selegiline, rasagiline, or safinamide on NMS or QOL using symptom-specific assessment batteries or objective measures, and (3) written in English. NMS included the following ones: depression, anxiety, sleep disturbances, fatigue, pain, autonomic dysfunctions, olfactory dysfunctions, cognitive dysfunctions, apathy, psychosis, impulse control disorders (ICD), and rapid eye movement sleep behavior disorders (RBD). Conference papers, review articles, and meta-analyses were excluded. “Real-world” studies were excluded because uncontrolled factors hindered the estimation of the impact of MAO-BIs. We reviewed the reference lists of included publications to find additional publications.

By using mean values and standard deviations (SD) at baseline (mean_T1_ and SD_T1_) and mean values after intervention (mean_T2_) for the scales of interest, we calculated effect sizes according to the following formula: Effect size = [(mean_T2_ − mean_T1_)/SD_T1_]. When the publications lacked required values, effect sizes were shown as “incalculable.” Based on the values, effect sizes were considered trivial (< 0.2), small (0.20–0.49), moderate (0.50–0.79), or large (≥0.8)^106^.

### Quality assessments

We assessed the study quality of the included studies using a PD-specific assessment form designed by den Brok et al., which was based on the Newcastle–Ottawa quality assessment scale (Supplementary Table 1)^107^.

## Supplementary information

Supplementary material

## Data Availability

All data relevant to the study are included in the article or uploaded as supplementary information.
