# Supplementary material for: Effects of MAO-B inhibitors on non-motor symptoms and quality of life in Parkinson’s disease: A systematic review
Source: NPJ Parkinsons Dis. 2022 Jun 13;8:75. doi: 10.1038/s41531-022-00339-2 (PMC9192747; doi:10.1038/s41531-022-00339-2)
Supplement: Supplementary file 1 — Supplementary material [file 41531_2022_339_MOESM1_ESM.pdf]

Supplementary material for "Effects of MAO-B inhibitors on non-motor symptoms and quality of life in Parkinson's disease: A Systematic Review" by Tsuboi et al.

Supplementary information 1: The search syntax for each database

Supplementary Table 1: Bias assessment form

Supplementary Table 2: Cognitive assessment outcomes of MAO-BI studies

Supplementary references: References for Supplementary s-3

Supplementary information 1: The search syntax for each database

1) Pubmed

((((Parkinson's disease[MeSH Terms]) OR (Parkinson disease[Title/Abstract])) OR (Parkinson's disease[Title/Abstract])) AND (((selegiline[Title]) OR (rasagiline[Title])) OR (safinamide[Title]))) AND (English[Language])

2) Embase

(TITLE (selegiline) OR TITLE (rasagiline) OR TITLE (safinamide)) AND (TITLE-ABS-KEY (parkinson's AND disease) OR TITLE-ABS-KEY (parkinson AND disease)) AND PUBYEAR > 2000 AND (LIMIT-TO (LANGUAGE , "English"))

3) Cochrane Library

#1 (Parkinson’s disease):ti,ab,kw or (Parkinson disease):ti,ab,kw

#2 rasagiline

#3 safinamide

#4 selegiline

#5 #2 OR #3 OR #4

#6 #1 AND #5

Supplementary Table 1: Bias assessment form

Author (year)	Selection bias						Confounding bias						Information bias			Selection bias	Confound ing bias	Informatio n bias	Total points
	Type of cohort	Recruitment	Reported exclusion criteria	%of patients included	Patient source	Diagnostic criteria of PD	Reported rate of depression	Reported on cognitive function	Reported on PD stage	Reported on disease duration	Reported on off stage	Reported on age	QOL/MNS assessment	Type of controls	Follow up period	＞B	＞B	＞C	
Parkinson study group (2005)	A	B	A	88%	A	A	A	A	A	A	A	A	B	A	A	5	6	2	13
Barone et al (2015)	A	B	A	94%	A	A	A	A	A	A	A	A	B	A	A	5	6	2	13
Hanagasi et al (2011)	A	B	A	91%	A	A	A	A	A	A	A	A	B	A	A	5	6	2	13
Schapira et al (2017)	A	B	A	89%	A	A	A	A	A	A	A	A	B	A	A	5	6	2	13
De Micco et al (2021)	A	A	A	100%	A	A	A	A	A	A	A	A	B	C	A	6	6	1	13
Dalrymple-Alford et al (1995)	A	A	A	100%	A	B	A	A	A	A	A	A	B	A	A	5	6	2	13
Parkinson study group (2002)	A	B	A	81%	A	A	A	C	A	A	A	A	B	A	A	5	5	2	12
Biglan et al (2006)	A	B	A	66%	A	A	A	C	A	A	A	A	B	A	A	5	5	2	12
Weintraub et al (2016)	A	B	A	89%	A	A	B	A	A	C	A	A	A	A	A	5	4	3	12
Zhang et al (2018)	A	B	A	85%	A	A	B	A	A	A	A	A	B	A	A	5	5	2	12
Stern et al (2004)	A	B	A	98%	A	A	A	B	A	A	A	A	B	A	A	5	5	2	12
Haehner et al (2013)	A	B	A	100%	A	A	C	A	A	A	C	A	A	A	A	5	4	3	12
Schrempf et al (2018)	A	B	A	83%	A	A	B	C	A	A	A	A	A	A	A	5	4	3	12
Schettino et al (2016)	A	B	A	100%	A	A	A	A	A	A	C	A	A	C	A	5	5	2	12
Stocchi et al (2012)	A	B	A	86%	A	B	A	A	A	A	A	A	B	A	A	4	6	2	12
Santos García et al (2021)	A	A	A	88%	A	A	A	B	A	A	A	A	B	C	A	6	5	1	12
Plastino et al (2021)	A	A	A	100%	A	A	C	A	A	A	C	A	A	C	A	6	4	2	12
Pålhagen et al (1998)	A	B	A	90%	A	B	A	A	A	A	A	A	B	A	A	4	6	2	12
Hattori et al (2018)	A	B	A	81%	A	A	C	B	A	A	A	A	B	A	A	5	4	2	11
Zang et al (2018)	A	B	A	92%	A	A	C	B	A	A	A	A	B	A	A	5	4	2	11
Hattori et al (2019)	A	B	A	86%	A	A	C	B	A	A	A	A	B	A	A	5	4	2	11
Hattori et al (2019)	A	B	A	70%	A	A	C	B	A	A	A	A	B	A	A	5	4	2	11
Lim et al (2015)	A	B	A	100%	A	A	A	B	B	A	C	A	A	A	A	5	3	3	11
Borgohain et al (2014)	A	B	A	89%	A	B	A	B	A	A	A	A	B	A	A	4	5	2	11
Borgohain et al (2014)	A	B	A	81%	A	B	A	B	A	A	A	A	B	A	A	4	5	2	11
Schapira et al (2013)	A	B	A	82%	A	B	A	A	A	C	A	A	B	A	A	4	5	2	11
Grigoriou et al (2021)	A	A	A	100%	A	B	A	B	A	A	A	A	B	C	A	5	5	1	11
Geroin et al (2020)	A	A	A	100%	A	A	B	A	A	A	B	A	B	C	A	6	4	1	11
Stocchi et al (2014)	A	B	A	97%	A	A	B	C	A	A	B	A	B	A	A	5	3	2	10
Hauser et al (2014)	A	B	A	88%	A	A	B	B	B	A	A	A	B	A	A	5	3	2	10
Frakey et al (2017)	A	B	A	90%	A	A	B	A	A	C	C	A	B	A	A	5	3	2	10
Hattori et al (2019)	A	B	A	73%	A	A	C	B	A	A	A	A	B	C	A	5	4	1	10
Rahimi et al (2016)	A	B	A	78%	A	A	A	A	B	A	C	A	B	C	A	5	4	1	10
Cattaneo et al (2017)	A	B	A	×	A	B	A	B	A	A	A	A	B	A	A	3	5	2	10
Hattori et al (2020)	A	B	A	86%	A	B	C	B	A	A	A	A	B	A	A	4	4	2	10
Tsuboi et al (2020)	A	B	A	70%	A	A	B	B	A	A	A	A	B	C	A	5	4	1	10
Allain et al (1991)	A	B	A	90%	A	B	A	B	A	B	A	A	B	A	A	4	4	2	10

Author (year)	Selection bias						Confounding bias						Information bias			Selection bias	Confounding bias	Information bias	Total points
	Type of cohort	Recruitment	Reported exclusion criteria	%of patients included	Patient source	Diagnostic criteria of PD	Reported rate of depression	Reported on cognitive function	Reported on PD stage	Reported on disease duration	Reported on off stage	Reported on age	QOL/MNS assessment	Type of controls	Follow up period	>B	>B	>C	
Cibulcik et al (2016)	A	B	A	95%	A	A	C	B	A	A	C	A	B	C	A	5	3	1	9
Rinaldi et al (2018)	A	A	A	86%	A	B	B	A	C	A	B	A	B	C	A	5	3	1	9
Tsuboi et al (2021)	A	B	A	×	A	B	C	B	A	A	A	A	B	A	A	3	4	2	9
Rinaldi et al (2021)	A	A	A	91%	A	B	B	A	C	A	B	A	B	C	A	5	3	1	9
Shoulson et al (1992)	A	B	A	×	A	B	A	B	A	B	A	A	B	A	A	3	4	2	9
Waters et al (2004)	A	B	A	94%	A	B	B	B	B	A	A	A	B	A	A	4	3	2	9
Hietanen et al (1991)	A	B	C	90%	B	B	A	A	A	A	C	A	B	A	A	2	5	2	9
Peña et al (2021)	C	B	A	95%	B	A	A	B	C	A	A	A	B	C	A	3	4	1	8
Panisset et al (2016)	A	B	C	88%	B	B	C	C	A	A	C	A	A	C	A	2	3	2	7
Müller et al (2013)	A	B	C	×	B	A	A	A	A	C	C	A	B	C	A	2	4	1	7
Brusa et al (2014)	A	B	C	×	B	A	C	C	A	A	C	A	A	C	A	2	3	2	7
Gómez-López et al (2021)	C	A	A	66%	B	B	C	B	A	A	C	A	B	C	A	3	3	1	7
Bianchi et al (2019)	C	B	C	100%	B	B	A	A	A	A	B	A	B	C	A	1	5	1	7
Cattaneo et al (2017)	A	B	A	×	A	B	A	B	C	C	B	C	B	A	A	3	1	2	6
Gallazzi et al (2021)	C	B	C	×	B	A	C	A	A	A	C	A	B	C	A	1	4	1	6
Cattaneo et al (2018)	A	B	A	×	A	B	C	C	C	C	B	C	B	A	A	3	0	2	5
Liguori et al (2018)	C	B	B	×	B	B	C	C	A	A	B	A	B	C	A	0	3	1	4
A	Prospective cohort	Consecutive	Reported	×: Insufficient infromation	Representative of PD community	Described	Fully	Fully	Fully	Disease duration	Fully	Fully	Scale compared to clinical assessment	Same community	Follow-up				
B	Cross sectional	Other/ not reported	Partially reported		Uncertain	Not described	In part	In part	In part	Duration of motor symptoms	In part	In part	Scale designed for specific symtroms	Different/ Unknown	No follow-up				
C	Retrospetive cohort		Not reported		Not representative		Not reported	Not reported	Not reported	Not reported	Not reported	Not reported	Other sale	Not reported					

Quality assessments of the included studies were performed the PD-specific assessment form designed by Den Brok et al. (Mov Disord 2015), which was based on the Newcastle–Ottawa quality assessment scale (Wells et al. The Newcastle-Ottawa Scale (NOS) for assessing the quality of nonrandomised studies in metaanalysis. [cited 2022 February 20]. Available from: http//www.ohri.ca/programs/clinical_epidemiology). The scores range from 0 to 15, and higher scores indicate better study quality.

Supplementary Table 2: Cognitive assessment outcomes of MAO-BI studies

Studies	Study design	Participants	Study quality	Age	Disease duration	Instruments	Outcome	Effect size
Hattori et al (2018)[1]	Multicenter, double-blind, placebo-controlled RCT, 26 weeks	404 patients, advanced PD with off time ≥ 2.5hours	1	66.1 (8.3)	9.0 (4.7)	PDQ-39: cognition	No significant difference between rasagiline 1mg and placebo, -0.91 (-4.40 to -2.58), p=0.6072	NA
						PDQ-39: cognition	No significant difference between rasagiline 0.5mg and placebo, 1.55 (-1.89 to 4.99), p=0.69	NA
Zang et al (2018)[2]	Multicenter, double-blind, placebo-controlled RCT, 16 weeks	324 patients, advanced PD with off time ≥ 1 hour	1	62.2 (9.4)	7.3 (4.6)	PDQ-39: cognition	No significant difference between rasagiline 1mg and placebo, -0.6 (-3.77 to 2.67), p=0.737	NA
Hauser et al (2014)[3]	Multicenter, double-blind, placebo-controlled RCT, 18 weeks	321 patients, early PD not adequately controlled with dopamine agonists	1	62.6 (9.7)	2.1 (2.1)	SCOPA-cognition	No significant differences between rasagiline 1 mg and placebo; statistics not shown	NA
Hattori et al (2019)[4]	Multicenter, double-blind, placebo-controlled RCT, 26 weeks	244 early PD patients not taking antiparkinsonian medication	1	66.4 (8.9)	1.8 (1.6)	PDQ-39: cognition	No significant differences between rasagiline 1mg and placebo; –1.15 (–2.88 to 2.22), P=0.7987	NA
Weintraub et al (2016)[5]	Multicenter, double-blind, placebo-controlled RCT, 24 weeks	170 patients, PD with mild cognitive impairment	1	67.6 (7.7)	NA	SCOPA-COG	No significant differences between groups; rasagiline 1mg 1.6±0.5 vs. placebo 0.8±0.5, P=0.22	0.32
						MoCA	No significant differences between groups; rasagiline 1mg 0.9±0.32 vs. placebo 1.0±0.34, P=0.84	0.56

						Brief Penn Daily Activities Questionnaire	No significant differences between groups; rasagiline 1mg –0.9±0.72 vs. placebo –0.1±0.75, P=0.48	0.09
Zhang et al (2018)[6]	Multicenter, double-blind, placebo-controlled RCT, 26 weeks	130 early PD patients not taking antiparkinsonian medication	1	59.0 (8.9)	0.1 (median)	PDQ-39: cognition	No significant differences between groups; rasagiline 1mg -1.97±1.87 vs. placebo 1.60±1.91, P=0.156	NA
Barone et al (2015)[7]	Multicenter, double-blind, placebo-controlled RCT, 12 weeks	123 patients, PD with moderate depression (BDI ≥ 15)	1	66.1 (8.5)	4.3 (12.5)	Aphasia Neuropsychological Examination	No significant difference between groups, rasagiline 1mg –0.68±2.85 vs. placebo –0.44±2.85, P value not shown	0.16
						RAVLT immediate recall	No significant difference between groups, rasagiline 1mg 2.27±10.79 vs. placebo –1.58±38.51, P value not shown	0.19
						RAVLT delayed recall	No significant difference between groups, rasagiline 1mg 0.92±2.27 vs. placebo 1.21±2.53, P value not shown	0.25
						Word reading Stroop test	No significant difference between groups, rasagiline 1mg -1.29±9.57 vs. placebo –0.92±13.64, P value not shown	0.07
						Color naming Stroop test	No significant difference between groups, rasagiline 1mg -1.65±5.85 vs. placebo 0.32±9.71, P value not shown	0.17
						Trail making test B–A	No significant difference between groups, rasagiline 1mg -4.22±53.03 vs. placebo 6.24±69.34, P value not shown	0.04

						Stroop test: non-congruent correct answers	No significant difference between groups, rasagiline 1mg 0.39±4.30 vs. placebo 0.58±13.18, P value not shown	0.05
						Clock Drawing Test	No significant difference between groups, rasagiline 1mg 0.33±3.40 vs. placebo –0.63±12.88, P value not shown	0.10
						Cognitive Performance Test for letter	No significant difference between groups, rasagiline 1mg -2.12±9.35 vs. placebo 0.36±7.18, P value not shown	0.15
						Cognitive Performance Test for categories	No significant difference between groups, rasagiline 1mg 1.71±10.86 vs. placebo 0.62±6.85, P value not shown	0.12
						Benton Judgment of Line Orientation Test	No significant difference between groups, rasagiline 1mg 0.98±3.73 vs. placebo 0.84±7.18, P value not shown	0.12
						Benton Judgment of Line Orientation Test: copy	No significant difference between groups, rasagiline 1mg -1.13±6.88 vs. placebo 0.07±11.21, P value not shown	0.17
						PDQ-39: cognition	Significantly better in rasagiline; rasagiline 1mg -4.00±2.28 vs. placebo 2.41±2.03, P=0.026	0.21
Hanagasi et al (2011)[8]	Multicenter, double-blind, placebo-controlled RCT, 12 weeks	55 patients, mild to moderate PD (HY stage 1–3) with mild cognitive impairment	1	66.4 (9.8)	4.0 (2.4)	Digit span: forward	No significant differences between rasagiline 1mg and placebo; 0.14±0.25, P=0.792	0.29

Digit span: backward	Significantly better in rasagiline 1mg vs placebo; 0.58±0.28, P=0.018	0.38
Digit span: total	No significant differences between rasagiline 1mg and placebo; 0.73±0.36, P=0.058	0.41
Digit Ordering Test	No significant differences between rasagiline 1mg and placebo; 3.79±1.9, P=0.052	0.30
Semantic verbal fluency	No significant differences between rasagiline 1mg and placebo; 2.17±141, P=0.06	0.30
Lexical verbal fluency	No significant differences between rasagiline 1mg and placebo; 2.62±1.81, P=0.156	0.31
Verbal fluency total	Significantly better in rasagiline 1mg vs placebo; 4.79±2.24, P=0.038	0.35
Clock drawing test	No significant differences between rasagiline 1mg and placebo; 0.48±0.66, P=0.489	0.17
Trail Making Test B-A	No significant differences between rasagiline 1mg and placebo; -2.50±32.14, P=0.939	0.07
Stroop time difference	No significant differences between rasagiline 1mg and placebo; 7.42±8.84, P=0.406	0.16
Stroop error	No significant differences between rasagiline 1mg and placebo; 0.38±2.08, P=0.47	0.12

Stroop spontaneous corrections	No significant differences between rasagiline 1mg and placebo; 1.89±1.02, P=0.056	0.16
Verbal immediate recall	No significant differences between rasagiline 1mg and placebo; -0.49±0.52, P=0.546	0.10
Verbal delayed free recall	No significant differences between rasagiline 1mg and placebo; -0.02±0.93, P=0.561	0.21
Verbal recognition	No significant differences between rasagiline 1mg and placebo; -0.51±0.89, P=0.573	0.11
Verbal delayed recall + recognition	No significant differences between rasagiline 1mg and placebo; -0.53±0.64, P=0.505	0.17
Verbal learning score	No significant differences between rasagiline 1mg and placebo; 0.24±4.42, P=0.956	0.11
Visual immediate recall	No significant differences between rasagiline 1mg and placebo; 0.48±0.73, P=0.514	0.29
Visual delayed recall	No significant differences between rasagiline 1mg and placebo; 0.98±0.90, P=0.283	0.68
Visual recognition	No significant differences between rasagiline 1mg and placebo; 0.11±0.40, P=0.501	0.39
Benton line orientation	No significant differences between rasagiline 1mg and placebo; 1.32±1.02, P=0.202	0.37

						Benton facial recognition	No significant differences between rasagiline 1mg and placebo; -0.31±1.50, P=0.835	0.31
						Boston Naming Test total score	No significant differences between rasagiline 1mg and placebo; 0.19±0.52, P=0.957	0.46
Frakey et al (2017)[9]	Single-center, double-blind, placebo-controlled RCT, 26 weeks	45 non-demented PD patients (MMSE > 21)	1	68.6 (8.0)	NA	Digit span: forward	No significant differences between groups; rasagiline 1mg, baseline 6.87±1.39 and post 6.61±1.23, placebo, baseline 7.0±1.15 and post 7.0±1.02, P value not shown	0.19
						Digit span: backward	No significant differences between groups; rasagiline 1mg, baseline 5.13±1.36 and post 4.79±1.08, placebo, baseline 4.41±1.15 and post 4.64±1.09, P value not shown	0.25
						Digit span: sequencing	No significant differences between groups; rasagiline 1mg, baseline 5.17±1.37 and post 5.52±1.12, placebo, baseline 5.68±1.13 and post 5.50±1.53, P value not shown	0.26
						Trail-Making Test, Part A	No significant differences between groups; rasagiline 1mg, baseline 32.22±8.66 and post 35.57±7.12, placebo, baseline 41.05±14.37 and post 43.32±22.19, P value not shown	0.39
						Trail-Making Test, Part B	No significant differences between groups; rasagiline 1mg, baseline 109.26±50.82 and post 114.74±54.19, placebo, baseline	0.11

	125.0±71.38 and post 116.82±79.02, P value not shown	
Clock	No significant differences between groups; rasagiline 1mg, baseline 8.78±1.17 and post 8.78±1.78, placebo, baseline 8.32±1.86 and post 8.55±1.74, P value not shown	0.00
Lexical verbal fluency	No significant differences between groups; rasagiline 1mg, baseline 36.35±11.13 and post 38.22±12.75, placebo, baseline 36.23±11.02 and post 37.5±13.27, P value not shown	0.17
Semantic verbal fluency	No significant differences between groups; rasagiline 1mg, baseline 18.48±4.05 and post 17.74±6.25, placebo, baseline 17.27±4.10 and post 17.77±4.92, P value not shown	0.18
Oral Symbol Digit Modalities Test	No significant differences between groups; rasagiline 1mg, baseline 43.48±8.61 and post 42.0±8.30, placebo, baseline 43.41±12.79 and post 41.64±13.71, P value not shown	0.17
Boston Naming Test	No significant differences between groups; rasagiline 1mg, baseline 26.87±2.47 and post 26.91±3.26, placebo, baseline 27.68±2.06 and post 27.86±2.21, P value not shown	0.02

						Repeatable Battery for the Assessment of Neuropsychological Status: line orientation	No significant differences between groups; rasagiline 1mg, baseline 16.17±3.05 and post 16.52±3.41, placebo, baseline 17.36±2.56 and post 16.64±2.85, P value not shown	0.11
						Rey Auditory Verbal Learning Test	No significant differences between groups; rasagiline 1mg, baseline 39.39±10.80 and post 38.43±11.54, placebo, baseline 38.86±9.36 and post 41.32±11.26, P value not shown	0.09
Hattori et al (2019)[10]	Multicenter, open-label, prospective, phase 3 study, 52 weeks	222 PD patients taking levodopa with or without motor fluctuation	3	68.0 (8.4)	7.1 (5.0)	PDQ-39: cognition	No significant change with rasagiline 1mg; baseline to post 0.03±16.38, P value not shown	NA
Cibulcik et al (2016)[11]	Single-center, open-label, prospective study, 3 months	42 patients, PD with freezing of gait	3	69.5 (7.9)	8.3 (4.3)	PDQ-39: cognition	No significant change with rasagiline 1mg; baseline 15.4±11.8 and post 15.7±12.0, p=0.834	0.03
Rahimi et al (2016)[12]	Single-center, open-label, prospective study, 90 days	14 patients, PD with freezing of gait	3	68.9 (6.7)	11.8 (5.0)	MoCA	No significant change with rasagiline 1 mg; mean values for the whole cohort not shown, P=0.91	NA
Rinaldi et al (2018)[13]	Single-center, open-label, prospective study, 16 weeks	14 patients, advanced PD with wearing off and MMSE ≥ 26	3	68.0 (6.0)	8.9 (2.8)	Frontal Assessment Battery	Significant improvement with rasagiline 1mg; baseline 11.1±3.1 and post 12.7±2.2, p<0.05	0.55

Borgohain et al (2014)[14]	Multicenter, double-blind, placebo-controlled RCT, 24 weeks	669 patients, advanced PD with off time ＞1.5 hours	1	59.9 (9.4)	8.1 (3.9)	PDQ-39: cognition	No significant differences between groups; safinamide 100mg –1.6 vs. placebo –0.5, P=0.3775	0.09
						PDQ-39: cognition	No significant differences between groups; safinamide 50mg –0.7 vs. placebo –0.5, P=0.3081	0.04
Schapira et al (2017)[15]	Multicenter, double-blind, placebo-controlled RCT, 24 weeks	549 patients, advanced PD with off time ＞1.5 hours	1	61.9(9.0)	8.9 (4.6)	MMSE	No significant differences between groups; safinamide 100mg –0.20±1.50 vs. placebo –0.05±1.61, P=0.26	0.14
						Cogtest PD Battery scores: Auditory No. sequencing	No significant differences between groups; safinamide 100mg -0.08±0.95 vs. placebo -0.03±0.19, P=0.01	0.09
						Cogtest PD Battery scores: Spatial working memory	No significant differences between groups; safinamide 100mg -0.21±4.11 vs. placebo 0.24±3.66, P=0.60	0.05
						Cogtest PD Battery scores: Strategic target detection	No significant differences between groups; safinamide 100mg 0.27±1.65 vs. placebo 0.13±1.61, P=0.44	0.20
						Cogtest PD Battery scores: Word list memory	No significant differences between groups; safinamide 100mg 0.21±1.45 vs. placebo 0.27±1.31, P=0.61	0.14
						Cogtest PD Battery scores: Symbol digit substitution	No significant differences between groups; safinamide 100mg 0.05±0.77 vs. placebo 0.14±0.68, P=0.24	0.06

						Cogtest PD Battery scores: Tower of London	No significant differences between groups; safinamide 100mg 0.17±1.03 vs. placebo 0.26±1.00, P=0.16	0.17
						Cogtest PD Battery scores: Word list memory delayed	No significant differences between groups; safinamide 100mg 0.09±1.55 vs. placebo 0.16±1.44, P=0.68	0.06
Stocchi et al (2012)[16]	Multicenter, double-blind, placebo-controlled RCT, 24 weeks	269 patients, early PD receiving a stable dose of a single dopamine agonist	1	57.4 (11.3)	2.5 (1.3)	MMSE	No significant difference between safinamide and placebo; statistical values not shown	NA
Schapira et al (2013)[17]	Multicenter, double-blind, placebo-controlled RCT, 18 months	227 patients, early PD taking a single dopamine agonist	1	median 56.6 and 59.8 for 100mg and 200mg	NA	MMSE	No significant differences between groups; safinamide 100 or 200mg 0.1±1.34 vs. placebo 0.4±1.69, P=0.297	0.07
Santos García et al (2021)[18]	Multicenter, open-label, prospective study, 6 months	50 patients, PD with non-motor burden (NMSS ≥ 40)	3	68.5 (9.1)	6.4 (5.1)	NMSS: Attention/memory	Significant improvement with safinamide 100mg; baseline 17.50±17.09 and post 13.32±18.19, p=0.026	0.24
						PDQ-39: cognition	No significant change with safinamide 100mg; baseline 27.2±22.0 and post 23.7±22.5, P=0.876	0.16
Rinaldi et al (2021)[19]	Single-center, open-label, prospective study, 12 weeks	35 patients, advanced PD with wearing off (WOQ-19 ≥ 3)	3	67.0 (8.0)	9.5 (3.0)	FAB	Significant improvement with safinamide 100mg; baseline 11.9±2.5 and post 13.6±2.1, P=0.0001	0.68

						Stroop Word-Color-Test: reading time (s)	No significant change with safinamide 100mg; baseline 15.7±2.7 and post 15.3±2.2, p=0.3038	0.15
						Stroop Word-Color-Test: reading errors	No significant change with safinamide 100mg; baseline 0.2±0.3 and post 0.3±0.4, p=0.8099	0.67
						Stroop Word-Color-Test: color naming time (s)	No significant change with safinamide 100mg; baseline 17.8±4.6 and post 17.2±3.1, p=0.1496	0.13
						Stroop Word-Color-Test: color naming errors	No significant change with safinamide 100mg; baseline 1.1±1.2 and post 0.6±0.2, p=0.0059	0.42
						Stroop Word-Color-Test: intereference time	Significant improvement with safinamide 100mg; baseline 40.9±8.7 and post 36.0±8.0, P=0.0001	0.56
						Stroop Word-Color-Test: interference errors	No significant change with safinamide 100mg; baseline 2.0±1.2 and post 1.6±1.5, p=0.0565	0.33
De Micco et al (2021)[20]	Single-center, open-label, prospective study, 6 months	20 patients, advanced PD with off time ＞ 1.5 hours	3	63.8 (10.2)	6.0 (2.2)	MoCA	No significant change with safinamide 50mg; baseline 22.0±3.27 and post 21.9±3.60, P=0.94	0.02
						PD-Cognitive Rating Scale	No significant change with safinamide 50mg; baseline 83.8±12.5 and post 88.0±14.4, P=0.37	0.34

Bianchi et al (2019)[21]	Single-center, open-label, retrospective study, 4.4 months	20 patients, advanced PD with motor fluctuations	4	75.0 (6.3)	14.5 (6.8)	MMSE	No significant change with safinamide 100mg; baseline 27.2±2.6 and post 26.4±4.7, P=0.60	0.31
Pålhagen et al (1998)[22]	Multicenter, double-blind, placebo-controlled RCT, 12 months	157 patients, early de novo PD	1	63.7 (8.0)	1.9 (1.5)	MMSE	No significant difference between groups; selegiline 10mg 0.5±1.2 vs. placebo 0.3±1.8, P value not shown	0.31
						MMSE	No significant difference between selegiline 10mg and placebo; mean values not shown, P = 0.74	NA
Dalrymple-Alford et al (1995)[23]	Single-center, double-blind, placebo-controlled RCT, 8 weeks	21 patients, early PD not taking antiparkinsonian medication	1	65.7 (9.2)	1.7 (1.7)	Rod orientation test	No significant difference between groups; selegiline 10mg, baseline 6.8±1.2 and post 5.9±1.6; placebo, baseline 7.1±3.0 and post 6.5±2.4, P value not shown	0.75
						Wisconsin card sorting task	No significant difference between groups; selegiline 10mg, baseline 68.0±19.8 and post 69.2±13.0; placebo, baseline 63.5±12.5 and post 70.1±19.3, P value not shown	0.06
						Rivermead behavioral memory test	No significant difference between groups; selegiline 10mg, baseline 18.6±2.9 and post 20.8±1.9; placebo, baseline 19.9±2.7 and post 20.2±2.6, P value not shown	0.76
						Advanced progressive matrices	No significant difference between groups; selegiline 10mg, baseline 5.2±3.3 and post 4.0±3.7; placebo, baseline 5.1±1.6 and post 4.5±2.8, P value not shown	0.36

Hietanen et al (1991)[24]	Single-center, double-blind, placebo-controlled RCT, 4 weeks	20 patients, early PD not taking levodopa	1	56.9 (8.9)	4.2 (2.2)	Wechsler Adult Intelligence Scale: similarities	No significant difference between groups; selegiline 30mg, baseline 17.9±4.1 and post 18.0±3.8, placebo, baseline 19.9±3.1 and post 20.2±3.0, P value not shown	0.02
						Wechsler Adult Intelligence Scale: block design	No significant difference between groups; selegiline 30mg, baseline 31.2±7.4 and post 33.6±5.6, placebo, baseline 33.2±7.8 and post 31.2±10.9, P value not shown	0.32
						Wechsler Memory Scale: digit span	No significant difference between groups; selegiline 30mg, baseline 9.2±1.1 and post 9.1±0.6, placebo, baseline 9.0±1.7 and post 9.7±1.2, P value not shown	0.09
						Wechsler Memory Scale: logical memory	No significant difference between groups; selegiline 30mg, baseline 9.7±3.4 and post 10.2±, placebo, baseline 9.0±3.1 and post 12.3±1.7, P value not shown	0.15
						Wechsler Memory Scale: associative learning	No significant difference between groups; selegiline 30mg, baseline 15.6±3.2 and post 17.3±3.0, placebo, baseline 15.6±3.6 and post 16.1±3.8, P value not shown	0.53
						Wechsler Memory Scale: visual reproduction	No significant difference between groups; selegiline 30mg, baseline 7.9±3.6 and post 8.0±3.4, placebo, baseline 9.0±3.9 and post 8.8±3.0, P value not shown	0.03

Visuospatial rotation (Mannequin test)	No significant difference between groups; selegiline 30mg, baseline 60.8±32.9 and post 58.2±35.8, placebo, baseline 53.9±25.6 and post 55.1±33.8, P value not shown	0.08
Trail making test B	No significant difference between groups; selegiline 30mg, baseline 159±71 and post 132±34, placebo, baseline 135±50 and post 180±126, P value not shown	0.38
Stroop test: part 2	No significant difference between groups; selegiline 30mg, baseline 56±18 and post 61±22, placebo, baseline 57±14 and post 57±13, P value not shown	0.28
Stroop test: part 3	No significant difference between groups; selegiline 30mg, baseline 123±39 and post 159±132, placebo, baseline 116±37 and post 111±40, P value not shown	0.92

Age and disease duration are presented as mean ± SD if available.

BDI = Beck Depression Inventory; FAB = Frontal Assessment Battery; HY stage Hoehn–Yahr stage; MMSE = Mini Mental State Examination; MoCA = Montreal Cognitive Assessment; NA = not assessed; NMSS = Non-Motor Symptoms Scale; PD = Parkinson’s Disease; PDQ = Parkinson's Disease Questionnaire; RAVLT = Rey Auditory Verbal Learning Test; RCT = Randomized Controlled Trial; SCOPA = SCales for Outcomes in PArkinson's disease; WOQ-19 = Wearing-Off Questionnaire-19

Supplementary references: References for Supplementary Table 2

1. Hattori N, Takeda A, Takeda S, et al. Efficacy and safety of adjunctive rasagiline in Japanese Parkinson's disease patients with wearing-off phenomena: A phase 2/3, randomized, double-blind, placebo-controlled, multicenter study. Parkinsonism Relat Disord 2018;53:21-27. doi:10.1016/j.parkreldis.2018.04.025
2. Zhang Z, Shao M, Chen S, et al. Adjunct rasagiline to treat Parkinson's disease with motor fluctuations: a randomized, double-blind study in China. Transl Neurodegener 2018;7(1):14. doi:10.1186/s40035-018-0119-7
3. Hauser RA, Silver D, Choudhry A, et al. Randomized, controlled trial of rasagiline as an add-on to dopamine agonists in Parkinson's disease. Mov Disord 2014;29(8):1028-1034. doi:10.1002/mds.25877
4. Hattori N, Takeda A, Takeda S, et al. Rasagiline monotherapy in early Parkinson's disease: A phase 3, randomized study in Japan. Parkinsonism Relat Disord 2019;60:146-152. doi:10.1016/j.parkreldis.2018.08.024
5. Weintraub D, Hauser RA, Elm JJ, et al. Rasagiline for mild cognitive impairment in Parkinson's disease: A placebo-controlled trial. Mov Disord 2016;31(5):709-714. doi:10.1002/mds.26617
6. Zhang Z, Wang J, Chen S, et al. Efficacy and safety of rasagiline in Chinese patients with early Parkinson's disease: a randomized, double-blind, parallel, placebo-controlled, fixed-dose study. Transl Neurodegener 2018;7(1):32. doi:10.1186/s40035-018-0137-5
7. Barone P, Santangelo G, Morgante L, et al. A randomized clinical trial to evaluate the effects of rasagiline on depressive symptoms in non-demented Parkinson's disease patients. Eur J Neurol 2015;22(8):1184-1191. doi:10.1111/ene.12724
8. Hanagasi HA, Gurvit H, Unsalan P, et al. The effects of rasagiline on cognitive deficits in Parkinson's disease patients without dementia: a randomized, double-blind, placebo-controlled, multicenter study. Mov Disord 2011;26(10):1851-1858. doi:10.1002/mds.23738
9. Frakey LL, Friedman JH. Cognitive Effects of Rasagiline in Mild-to-Moderate Stage Parkinson's Disease Without Dementia. J Neuropsychiatry Clin Neurosci 2017;29(1):22-25. doi:10.1176/appi.neuropsych.15050118
10. Hattori N, Takeda A, Takeda S, et al. Long-term safety and efficacy of adjunctive rasagiline in levodopa-treated Japanese patients with Parkinson's disease. J Neural Transm 2019;126(3):289-297. doi:10.1007/s00702-018-1962-5
11. Cibulcik F, Benetin J, Kurca E, et al. Effects of rasagiline on freezing of gait in Parkinson's disease - an open-label, multicenter study. Biomed Pap Med Fac Univ Palacky Olomouc Czech Repub 2016;160(4):549-552.

doi:10.5507/bp.2016.023
12. Rahimi F, Roberts AC, Jog M. Patterns and predictors of freezing of gait improvement following rasagiline therapy: A pilot study. Clin Neurol Neurosurg 2016;150:117-124. doi:10.1016/j.clineuro.2016.08.025
13. Rinaldi D, Assogna F, Sforza M, Tagliente S, Pontieri FE. Rasagiline for dysexecutive symptoms during wearing-off in Parkinson's disease: a pilot study. Neurol Sci 2018;39(1):141-143. doi:10.1007/s10072-017-3123-2
14. Borgohain R, Szasz J, Stanzione P, et al. Randomized trial of safinamide add-on to levodopa in Parkinson's disease with motor fluctuations. Mov Disord 2014;29(2):229-237. doi:10.1002/mds.25751
15. Schapira AH V, Fox SH, Hauser RA, et al. Assessment of Safety and Efficacy of Safinamide as a Levodopa Adjunct in Patients With Parkinson Disease and Motor Fluctuations: A Randomized Clinical Trial. JAMA Neurol 2017;74(2):216-224. doi:10.1001/jamaneurol.2016.4467
16. Stocchi F, Borgohain R, Onofrj M, et al. A randomized, double-blind, placebo-controlled trial of safinamide as add-on therapy in early Parkinson's disease patients. Mov Disord 2012;27(1):106-112. doi:10.1002/mds.23954
17. Schapira AH V, Stocchi F, Borgohain R, et al. Long-term efficacy and safety of safinamide as add-on therapy in early Parkinson's disease. Eur J Neurol 2013;20(2):271-280. doi:10.1111/j.1468-1331.2012.03840.x
18. Santos García D, Labandeira Guerra C, Yáñez Baña R, et al. Safinamide Improves Non-Motor Symptoms Burden in Parkinson's Disease: An Open-Label Prospective Study. Brain Sci 2021;11(3):316. doi:10.3390/brainsci11030316
19. Rinaldi D, Sforza M, Assogna F, et al. Safinamide improves executive functions in fluctuating Parkinson's disease patients: an exploratory study. J Neural Transm 2021;128(2):273-277. doi:10.1007/s00702-020-02259-y
20. De Micco R, Satolli S, Siciliano M, et al. Effects of safinamide on non-motor, cognitive, and behavioral symptoms in fluctuating Parkinson's disease patients: a prospective longitudinal study. Neurol Sci May 2021. doi:10.1007/s10072-021-05324-w
21. Bianchi MLE, Riboldazzi G, Mauri M, Versino M. Efficacy of safinamide on non-motor symptoms in a cohort of patients affected by idiopathic Parkinson's disease. Neurol Sci 2019;40(2):275-279. doi:10.1007/s10072-018-3628-3
22. Pålhagen S, Heinonen EH, Hägglund J, et al. Selegiline delays the onset of disability in de novo parkinsonian patients. Swedish Parkinson Study Group. Neurology 1998;51(2):520-525. doi:10.1212/wnl.51.2.520
23. Dalrymple-Alford JC, Jamieson CF, Donaldson IM. Effects of selegiline (deprenyl) on cognition in early Parkinson's disease.

Clin Neuropharmacol 1995;18(4):348-359. doi:10.1097/00002826-199508000-00007

24. Hietanen MH. Selegiline and cognitive function in Parkinson's disease. Acta Neurol Scand 1991;84(5):407-410. doi:10.1111/j.1600-0404.1991.tb04978.x
